# Similarities between *Ciona* Dorsal Motor Ganglion and Vertebrate Cerebellum: Did a Chordate Ancestor Already Show D/V Subdivision within a Hindbrain Precursor?

**DOI:** 10.1523/ENEURO.0362-25.2026

**Published:** 2026-08-19

**Authors:** Matthew J. Kourakis, Yishen Miao, Erin D. Newman-Smith, Kerrianne Ryan, William C. Smith

**Affiliations:** ^1^Neuroscience Research Institute, University of California Santa Barbara, Santa Barbara, California 93106; ^2^Department of Molecular, Cellular and Developmental Biology, University of California, Santa Barbara, California 93106; ^3^Life Sciences Centre, Dalhousie University, Halifax, Nova Scotia B3H 1A5, Canada

**Keywords:** cerebellum, chordate, *Ciona*, evolution, hindbrain

## Abstract

The cerebellum, a major structure of the vertebrate central nervous system, forms the dorsal region of the hindbrain. While evidence from connectomics, neuron classification, and gene expression analyses suggests that the major CNS divisions of forebrain, midbrain, hindbrain, and spinal cord predate the vertebrate/tunicate divergence, it is unknown if the subdivision of the hindbrain into a cerebellum, or cerebellum precursor structure, does as well. In fact, conflicting views exist over whether even the most basal vertebrates, the cyclostomes, have a cerebellum homolog. We report here that the motor ganglion (MG), a structure that has been homologized to the vertebrate hindbrain in the chordate *Ciona* (a tunicate), has functionally distinct dorsal and ventral domains, similar to the generalized hindbrain of vertebrates. In *Ciona,* the dorsal MG functions as an ascending relay and processing center for peripheral sensory neurons, while the ventral domain processes and relays descending sensorimotor commands. These differences are reflected in distinct neuron classes present in the two domains, as well as in gene expression. While the dorsal MG of *Ciona* and HB of vertebrates differ in function and complexity, they share a number of similarities. Thus, while it is likely that the cerebellum proper evolved within vertebrates, our findings support the hypothesis that a simpler, dorsally positioned antecedent was present before the tunicate–vertebrate divergence.

## Significance Statement

 Investigation of vertebrates and their chordate relatives, the tunicates and cephalochordates, has identified conserved features in their respective central nervous systems that provide insight into their last common ancestor, an early representative of the chordates. Making inferences from studies of the tunicate model *Ciona*, we propose a scenario supporting the early appearance in chordates of a simple dorsal hindbrain structure, anatomically and functionally discrete, predating the cerebellum, which appeared only later in the vertebrate lineage. The data we consider span anatomy, development, function, gene expression, and, critically, neural circuitry. While the fully formed cerebellum is an innovation unique to vertebrates, we propose its origins can be traced to an ancient chordate that already had a dorsoventrally subdivided hindbrain/motor ganglion region.

## Introduction

**Figure 1.**
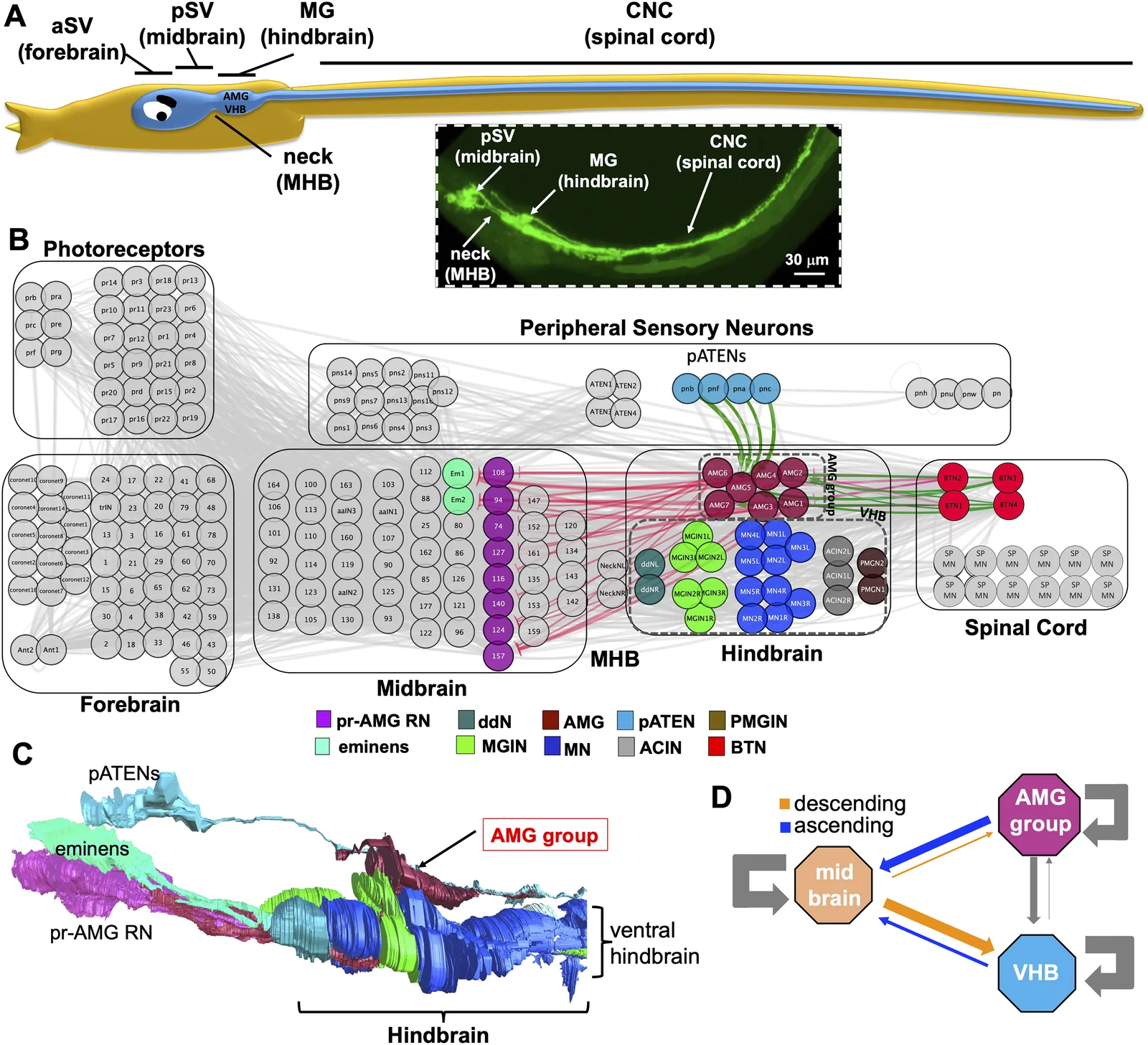
The *Ciona* larval hindbrain has distinct dorsal and ventral domains. ***A***, Diagram of a *Ciona* larva highlighting the central nervous system (blue). CNS domains are labeled with their traditional tunicate names and their putative vertebrate homologs (parentheses). Inset shows major larval CNS subdivisions (excluding the forebrain) labeled with GFP driven by the *cis*-regulatory region of the VACHT gene. ***B***, The entire *Ciona* larval CNS connectome highlighting the neurons of the hindbrain, as well as their primary presynaptic inputs (pATENs and BTNs), and postsynaptic targets (eminens and pr-AMG RNs). Green lines indicate putative excitatory synapses, and red lines indicate putative inhibitory synapses. Color coding of neurons is according to Ryan and Meinertzhagen (2019). ***C***, Lateral view of the hindbrain reconstructed from serial-section electron micrographs. Also shown are primary inputs to the AMGs, the pATENs, as well as the targets of the AMGs, the pr-AMG RNs, and eminens cells. ***D***, Summary of the synaptic connectivity between the AMG group, the ventral hindbrain and the midbrain. The strengths of all synaptic connections between neurons in the brain regions were summed and then normalized by the number of neurons in each region. These values are reflected in the width of the arrows between the brain regions. aSV, anterior sensory vesicle; pSV, posterior sensory vesicle; MHB, midbrain-hindbrain boundary; MG, motor ganglion; AMG, Ascending MG interneuron; VHB, ventral hindbrain; CNC, caudal nerve cord; pr-AMG RN, photoreceptor-ascending motor ganglion relay neuron; ddN, descending decussating neuron; MGIN, motor ganglion interneuron; MN, motor neuron; pATEN, posterior apical trunk epidermal neuron; ACIN, ascending contralateral inhibitory neuron; PMGIN, posterior MG interneuron; BTN, bipolar tail neuron. See Extended Data Figure 1-1.

The tadpole larva of the tunicate *Ciona* is the most widely studied of invertebrate chordate models, and is the only chordate with a published connectome (Ryan et al., 2016). The fully developed *Ciona* larva, at ∼1 mm in length, is small relative to larvae of vertebrates (e.g., amphibians and fish), having only ∼2,600 cells, of which ∼180 comprise the central nervous system (Ryan and Meinertzhagen, 2019). Despite its diminutive size, the *Ciona* larval CNS drives a complex set of behaviors (Zega et al., 2006; Salas et al., 2018; Bostwick et al., 2020). The accumulation of data on the *Ciona* larval CNS points to likely correspondence with major CNS subdomains of vertebrates (Fig. 1*A*; Hudson, 2016; Kourakis et al., 2025). In recent decades, much of the investigation of chordate CNS evolution focused on uncovering homologies based on gene expression. For example, Wada et al. and Imai et al. examined possible forebrain, midbrain, hindbrain, and spinal cord homology for *Ciona* and vertebrate CNS subdomains based on similarities in expression for select transcription factors and genes encoding signaling molecules (Wada et al., 1998; Imai et al., 2002). For the *Ciona* larval CNS, most expression patterns are supportive of the homologies shown in Figure 1*A*, although the anomalous expression of several genes leads to alternative interpretations. For example, the anterior domain of the *Ciona* larval CNS, called the sensory vesicle (SV; also known as the brain vesicle; Fig. 1*A*), is divided into anterior and posterior regions (aSV and pSV) that arise from different eight-cell stage lineages (Hudson, 2016). The expression of a number of genes, including Otx, DMRT, and Lhx2 in the aSV and Pax3/7, FoxB, and Otx in the pSV, points to the aSV being homologous to the forebrain and the pSV being homologous to the midbrain [reviewed in Borba et al. (2021)]. However, the absence of Dmbx expression in the pSV, a gene important for vertebrate midbrain development, brought into question the presence of a midbrain in *Ciona* (Takahashi and Holland, 2004). Nevertheless, the accumulation of evidence supports homology between the pSV and the midbrain, including, significantly, more recently described connectomic data which show that the pSV uniquely receives, processes, and integrates multimodal sensory inputs (Kourakis et al., 2019; Bostwick et al., 2020; Borba et al., 2021; Chung et al., 2023), paralleling the function to the vertebrate midbrain tectum.

Posterior to the pSV is an anatomical constriction in the neural tube called the neck (Fig. 1*A*), which, based on anatomy, gene expression, and function, is thought to share homology to the vertebrate midbrain-hindbrain boundary (MHB; Takahashi and Holland, 2004; Imai et al., 2009). Further posterior in the *Ciona* larval CNS are two domains: the first is a thickening in the neural tube called the motor ganglion (MG; also formerly, the visceral ganglion), and the second is a narrow and elongated domain extending posteriorly from the MG, running the length of the tail, called the caudal nerve cord (CNC; Fig. 1*A*). By anatomy alone, these two domains would appear to correspond to the vertebrate hindbrain and spinal cord, respectively. However, several observations brought into question their anatomical homology to vertebrate structures. It was only recently demonstrated that the *Ciona* CNC contains motor neurons (Kourakis et al., 2025); the previously perceived lack of motor neurons in the CNC had led to speculation that the MG instead corresponds to both the hindbrain and the spinal cord—despite the obvious morphological incongruence (Dufour et al., 2006). Further complicating the picture, *Ciona* lacks a member of the Gbx family of homeobox transcription factors (Wada et al., 2003), which in vertebrates play critical roles in the development of the MHB and anterior hindbrain, leading to the proposal that *Ciona* larvae may lack a hindbrain (Goode and Elgar, 2009; Nakayama et al., 2013). Similar to the pSV, the accumulated evidence for homology, including neuron types within the MG and their synaptic connectivity, supports hindbrain homology (Ryan et al., 2017; Kourakis et al., 2025). For example, neurons in the MG include an anterior pair, the descending decussating neurons (ddNs), which have circuit homology to hindbrain Mauthner cells of fish and amphibians (Ryan et al., 2017). Additionally, a prominent component of the MG is six pairs of motor ganglion interneurons (MGINs), which receive descending motor commands from the pSV and in turn project to motor neurons in the MG and CNC (Ryan et al., 2016; Kourakis et al., 2025). The connectivity and evident function of the MGINs, as well as their expression of the gene Vsx (Kourakis et al., 2021), support their homology to vertebrate hindbrain reticulospinal neurons (RSNs). These data are summarized in Extended Data Figure 1-1. Thus, anatomy, function, neuron class, synaptic connectivity, and, to a varying degree, gene expression support homology between the *Ciona* MG and CNC to the vertebrate hindbrain and spinal cord, respectively (Ryan et al., 2017; Kourakis et al., 2021, 2025; Kim et al., 2025). It should be noted that gene expression can be misleading in identifying common origins of apparently homologous tissues, as is observed, for example, in the divergent gene regulatory networks specifying notochords of among the tunicates (Kugler et al., 2011).

We present here new and published data showing that the *Ciona* motor ganglion, a prospective hindbrain homolog, has distinct dorsal and ventral domains. The dorsal and ventral subdomains of the *Ciona* hindbrain are characterized by their distinctly different developmental origins, gene expression, neuron classes, and function. This raises the question as to whether the dorsal/ventral subdivision of the motor ganglion/hindbrain originated independently in the vertebrates and tunicates, or alternatively, was present in their common ancestor. The presumed function and relative simplicity of the dorsal MG of *Ciona* (Ryan et al., 2018) indicate that it is not a cerebellum, consistent with previous speculation that the cerebellum arose within the vertebrates. However, we report that it shares many features with the dorsal domain of the vertebrate hindbrain, including induction by FGF, extensive gene expression conservation, and core circuit architecture. These observations lead us to hypothesize that the subdivision of the hindbrain into functional dorsal and ventral domains preceded the divergence of tunicates and vertebrates and that therefore the precursor of the cerebellum was already present at the base of the vertebrate lineage. In addition to the inferences from *Ciona*, we discuss how examination of organisms which stand in for key branches of chordate phylogeny may help to clarify details of major transitions in chordate hindbrain and cerebellar evolution; these organisms include the cephalochordate amphioxus, whose lineage diverged at the base of the chordates, and the agnathan vertebrate lamprey.

## Materials and Methods

### Animals

Adult *Ciona robusta* (aka, *Ciona intestinalis* type A) were collected at the Santa Barbara Harbor with the exception of the stable VGAT > Kaede line (Tg[MiCiVGATK]2, which was obtained from CITRES https://marinebio.nbrp.jp/ciona/) and cultured at the UC Santa Barbara Marine Laboratory. Larvae were generated by dissecting gametes from adults. VGAT > Kaede transgenic larvae were produced by crossing wild-type eggs with frozen VGAT > Kaede sperm aliquots.

### HLH-2 expression construct

A 1.5 kb genomic upstream sequence of the HLH-2 (KY21.Chr4.865; Satou et al., 2022) gene was amplified using the primers 5′-CCTGCAGGTCGACTCTAGAGAATGTGAACGCTCCAGATG*-3*′ and 5′-TCGGAGGAAGCCATTGGTACACTCATGTTGATAGAAGTTTGTATAAAAC*-3*′. NEBuilder HiFi Assembly (New England Biolabs) was used to join the promoter region and the first four amino acids in frame to a *Ciona* optimized RFP vector (pSP72-1.27 mRFPci; Zeller et al., 2006).

### In situ hybridization

To label mRNA fluorescently in whole-mount *Ciona* larvae or embryos, the hybridization chain reaction (HCR) in situ method (Molecular Instruments) was followed, as previously described (Kourakis et al., 2019). Genes for which probe sets were designed and their sequence identifiers are as follows: vesicular acetylcholine transporter (VACHT; NM_001032789.1), motor neuron homeobox (MNX; KH2012:KH.L128.12), lim-domain homeobox 1/5 (LHX/5; KH2012.L107.7), vesicular GABA transporter (VGAT; NM_001032573.1), and Orthopedia (OTP; XM_009862516.3).

### Immunolabeling

Labeling with antibodies was performed as previously described (Bostwick et al., 2020). Primary antibodies used were as follows: mCherry (rat; Life Technologies, M11217), Kaede (rabbit; MBL, PM012M), and GFP (mouse; Invitrogen, A11120). Appropriate fluorescent Alexa Fluor secondary antibodies were purchased from Invitrogen (including AF 488, A10680; AF 594, A11412; AF488, A11006).

### SU5402 treatment

SU5402 (Sigma, 572630) was diluted in DMSO to make a stock solution at 10 µg/µl, which was diluted to a working solution ranging from 20 to 40 µM. DMSO-only served as a negative control. The drug was added to seawater-filled dishes containing late neurula-stage embryos, which had resulted from a cross between wild-type eggs and stable transgenic VGAT > Kaede sperm. Thirty minutes after addition of the drug, the embryos were washed four times with filtered seawater, then allowed to reach the larval stage, when they were fixed for immunolabeling, and then prepared for confocal imaging.

### Ciona connectome

Analysis of *Ciona* connectomic data and generation of connectivity diagrams were performed using Cytoscape (Shannon et al., 2003). The connectivity matrix is available for download from (Ryan et al., 2016). Reconstruction of *Ciona* CNS domains using serial-section EM images from the connectome project was generated using the Cionectome Viewer on Morphonet (https://morphonet.org/).

### Phylogenetic analysis

Protein–protein BLAST was performed using human Ptf1a (NP_835455.1) in the NCBI nonredundant protein sequences database. Eighteen proteins in nine diverse chordate taxa were selected for phylogenetic analysis (mouse ptf1a, NP_061279.2; chicken ptf1a, XP_015137641.1; frog ptf1a, NP_001167491.1; hagfish ptf1a, BCS79892.1; lancelet ptf1a, CAH1239013.1; lancelet fer3-like, CAH1267229.1; lamprey ptf1a, XP_032824760.1; human fer3-like, EAW93713.1; mouse fer3-like, NP_277057.1; chicken fer3-like, XP_040520525.1; frog fer3-like, XP_018122450.1; *Ciona* ptf1a-related, XP_026693718.1; *Styela* fer3-like, XP_039261045.1; human twist-related, CAA67664.1; mouse twist-related, NP_035788.1; frog twist-related, NP_001079352.1; lamprey twist-related, XP_032807567.1).

The evolutionary history was inferred by using the maximum likelihood method and Jones–Taylor–Thornton (JTT) matrix-based model with 500 bootstrap replicates (Jones et al., 1992). Initial tree for the heuristic search was obtained automatically by applying neighbor-joining and BioNJ algorithms to a matrix of pairwise distances estimated using the JTT model and then selecting the topology with superior log likelihood value. Evolutionary analyses were conducted in MEGA11 (Tamura et al., 2021).

### Transgenesis by electroporation

Plasmids Hox10 > GFP (gift from Stolfi lab), ASIC> and NSCL-2 > GFP reporter constructs, and VGAT > jGCaMP6f (Chen et al., 2013) were electroporated into one-cell stage embryos, as described previously (Zeller, 2018). Briefly, fertilized eggs were first dechorinated with 1% sodium thioglycolate and 0.05% protease E and then washed extensively with seawater. The dechorionated fertilized eggs in 300 µl of seawater were mixed with 50 µg DNA in 480 µl 0.77 M mannitol solution and electroporated at 0.05 kV. Fertilized eggs were then washed extensively with seawater and cultured at 18°C in seawater with 0.1% methyl cellulose. Resulting larvae were either fixed for immunolabeling as described previously (Kourakis et al., 2021) or imaged in vivo (see below).

### GCaMP imaging

At the larval stage, transgenics expressing for VGAT > jGCaMP6f were mounted under coverslips raised with ∼80 µm spacers on glass slides in filtered seawater at 24–26 h post fertilization, as described previously (Chung et al., 2023). GCaMP6f fluorescence was recorded with a Leica DM6B fluorescence microscope using a 20× lens at a frame rate of 10–15 frames per second. The resulting movies were analyzed for temporal changes in fluorescence in selected regions of interest corresponding to the AMG cells using ImageJ (Abramoff et al., 2004). Normalized fluorescence values (Δ*F*/*F*_0_) were calculated as previously described (Chung et al., 2023).

### Single-cell RNA sequencing analysis

The raw sequencing reads of two previously published larval datasets (SRP198321 and SRP166883) were retrieved from Sequence Read Archive (Cao et al., 2019; Sharma et al., 2019). The reads were mapped to the KY2021 reference genome using Cell Ranger 8.0.1 (Zheng et al., 2017; Satou et al., 2019). The datasets were integrated with a linearly decoded variational auto-encoder (LDVAE; Svensson et al., 2020). The optimal configuration of the hyperparameters of the LDVAE was chosen by sweeping the hyperparameter space. Leiden clustering was performed using ScanPy (Wolf et al., 2018). Differential gene expression analysis was performed using scvi-tool 1.2 (Gayoso et al., 2022). The name of the closest human BLAST result (*e*-value < 0.05) from the UniProt database was used to annotate the KY21 genes.

### Cross-species analysis

One-to-one protein orthology between the mouse M10 genome (GRCm38.p4) and the *Ciona* KY2021 genome was identified using OrthoFinder (Emms and Kelly, 2019). The mouse cerebellum datasets were mapped to the KY2021 genome using ortholog genes as previously described (Kozareva et al., 2021; Zhong et al., 2025). The transformed mouse datasets were integrated with the *Ciona* dataset generated in the previous section using scvi-tool 1.2 and ScanPy. Mean dot products between the expression vector in the LDVAE latent space were calculated for every possible pair of *Ciona* and mouse clusters.

### Data and materials availability

The integrated *C. robusta* larva-stage single-cell RNA sequencing dataset and the complete differential expression data for all clusters shown in Figure 4*C* are deposited at Zenodo (https://doi.org/10.5281/zenodo.15320250).

## Results

### The *Ciona* larval motor ganglion has distinct dorsal and ventral domains

The following description of neuronal connectivity in the *Ciona* larva is derived from the connectomic work of Ryan et al. (2016). Here, as previously described (Chung et al., 2023; Borba et al., 2024), we will use interchangeably the names of the *Ciona* CNS domains and their corresponding vertebrate homologs [i.e., the “motor ganglion” (MG) may be referred to also as the “hindbrain”]. Figure 1*A* shows the correspondences. The *Ciona* larval connectome project identified a total of 30 neurons of six different types in the hindbrain [color coded in Fig. 1*B* according to Ryan and Meinertzhagen (2019)]. These neurons have been identified by their position, morphology, connectivity (Ryan et al., 2016), and, in some cases, gene expression (Gibboney et al., 2020) or neurotransmitter use (Kourakis et al., 2019). Figure 1*B* shows the *Ciona* larval MG in the context of the entire chemical synaptic connectome in which all neurons of the MG are highlighted (colorized). Also labeled are four peripheral sensory neurons called the posterior apical trunk epidermal neurons (pATENs), which are presynaptic to the AMGs (green arrows), and a second major class of input neurons to the AMGs, the bipolar tail neurons (BTNs). The primary targets of the AMGs are in the pSV/midbrain and consist almost exclusively of the eight photoreceptor-ascending MG neuron relay neurons (pr-AMG RNs) and the two eminens neurons. A reconstruction of the *Ciona* larval MG/hindbrain from the serial EM sections of the connectome project shows that it is composed of distinct ventral and dorsal domains that differ in cell types, connectivity, and lineage. The dorsal MG/hindbrain is made up exclusively of the AMG neurons (called henceforth the AMG group; Fig. 1*C*). The AMG group consists of six inhibitory/VGAT-positive interneurons (AMGs 1,2,3,4,6, and 7), surrounding a central excitatory/VACHT-positive neuron (AMG5; Ryan et al., 2018; Kourakis et al., 2019). Neurons of the AMG group also likely derive from B lineages (Cole and Meinertzhagen, 2004; Popsuj and Stolfi, 2021), not the A lineage as for neurons of the MG (Nishitsuji et al., 2012).

10.1523/ENEURO.0362-25.2026.f1-1 Figure 1-1 Summary of key similarities between vertebrate and tunicate (*Ciona*) central nervous systems. Download Figure 1-1, TIF file.

Connectome data show that the pATENs project exclusively to the AMGs, leading to their identification as a peripheral sensory relay center (Ryan et al., 2018). The AMGs in turn send ascending projections to the putative midbrain pr-AMG RNs and eminens cells (Fig. 1*B*; Ryan et al., 2018). In contrast to the dorsal/AMG domain, the ventral hindbrain/MG domain of *Ciona* receives and executes sensorimotor commands from descending excitatory and inhibitory relay interneurons of its midbrain (Kourakis et al., 2019). The primary midbrain synaptic targets in the hindbrain are the RSN-like MGINs, which in turn project to motor neurons in the hindbrain and spinal cord (Ryan et al., 2016; Kourakis et al., 2025). Also in the *Ciona* larval ventral hindbrain are the ddNs, as well as the ascending contralateral inhibitory neurons (ACINs), and the two posterior MG interneurons (PMGINs). The ACINs, VGAT^+^/glycinergic, in contrast to the VGAT^+^/GABAergic neurons of the dorsal AMG (Nishino et al., 2010), are thought to be components of the swimming central pattern generator (Horie et al., 2010), while the function(s) of the PMGINs is unknown. The distinction between functions of the AMG group and the ventral hindbrain is particularly evident when their connectivities to each other, and to the midbrain, are compared (Fig. 1*D*). While the ventral hindbrain receives extensive descending input from the midbrain, the AMG group receives very little and is instead characterized by its extensive ascending projections to the midbrain, consistent with its role as a relay center for peripheral sensory inputs. While there is extensive synaptic connectivity within the AMG group and the ventral hindbrain (Fig. 1*D*, gray arrows), there is very little between the two and the few synaptic connections that are present consist almost exclusively of projections from the AMG group to the ventral hindbrain.

### The AMG group is predicted to process mechanosensory input

**Figure 2.**
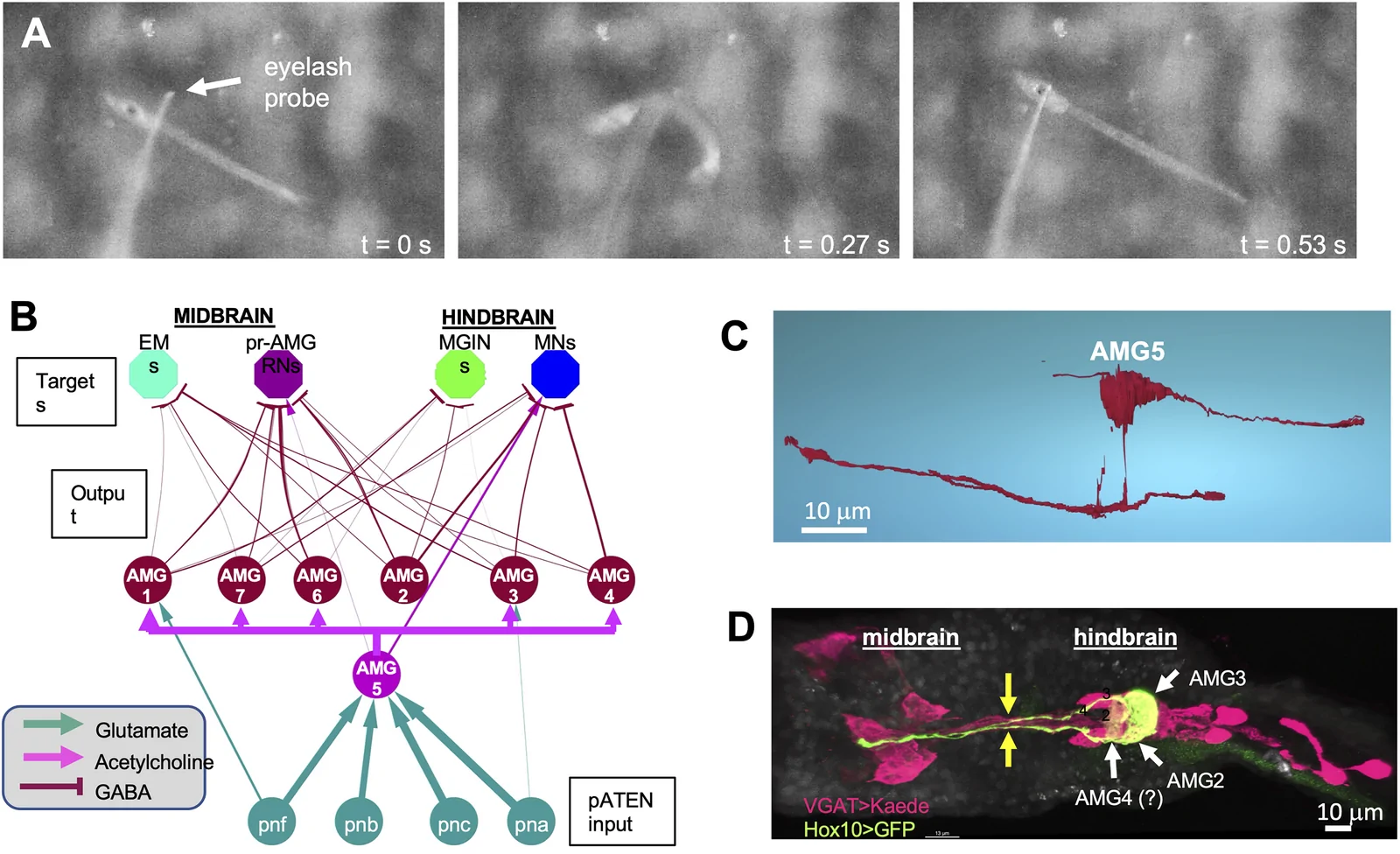
Activity and circuitry of the AMG group*.* ***A***, Response of a *Ciona* larva to mechanical stimulation (i.e., touch) of the pATENs. s, seconds. *Ciona* larvae are ∼1 mm in length. See Extended Data Movie 1*B*, neural circuit of the AMG group. ***B***, Input from the mechanosensory pATENs (*pnf*, *pnb*, *pnc*, and *pna*) primarily target AMG5 (cholinergic), which then branches to inhibitory output AMG neurons. ***C***, Reconstruction of AMG5 from serial-section electron microscopy. ***D***, Ascending projections from AMGs 2 and 3 labeled with a Hox10 promoter > GFP plasmid (green). Also shown are VGAT-positive neurons (magenta). Faint green fluorescence observed anterior to AMG2 and 3 may be AMG 4 (see Fig. 1 legend for abbreviations). Dorsal view is shown; anterior is left.

Epidermal sensory neurons (e.g., the pATENs) in *Ciona* larvae are thought to be either mechanosensitive, chemosensitive, or both (Ryan et al., 2018). In an effort to integrate connectomic information with our own manipulations of larval behavior, we observed that touching the dorsal region containing the pATENs of a larva with a fine probe resulted in an evoked swim, consistent with their being mechanosensory, although they are also reported to be chemosensory (Hoyer et al., 2024; Fig. 2*A*; three additional examples are shown in Movie 1). The four pATENs are glutamatergic (Kourakis et al., 2019) and project almost exclusively to the sole excitatory AMG, AMG5 (Fig. 2*B*; Ryan et al., 2018; Kourakis et al., 2019). AMG5 has a large bifurcating axon (Fig. 2*C*) not found in the other AMGs [see Source data 1, Fig. 1 of Ryan et al. (2016) for serial-section EM reconstructions of all larval neurons and Popsuj and Stolfi (2021) for fluorescent light microscopic imaging of AMG5] and is presynaptic to the inhibitory AMGs, with the apparent exception of AMG2 (Fig. 2*B*; Ryan et al., 2016). The connectome shows that output from the AMG group to the midbrain is via the ascending projections of the inhibitory AMGs (Fig. 2*B*). Using a Hox10 *cis*-regulatory region driving GFP (*25*), we find that the ascending axons of AMG2 and 3 are evident (Fig. 2*D*, yellow arrows). Thus, the AMG group changes the valence of the mechanosensory input of the pATENs from excitatory (glutamate via acetylcholine) to outgoing inhibitory (GABA).

**Movie 1.** pATENs are mechanosensitive. Three *Ciona* larvae respond to being touched at pATENs with a fine probe. The movie plays in real time. [View online]

**Figure 3.**
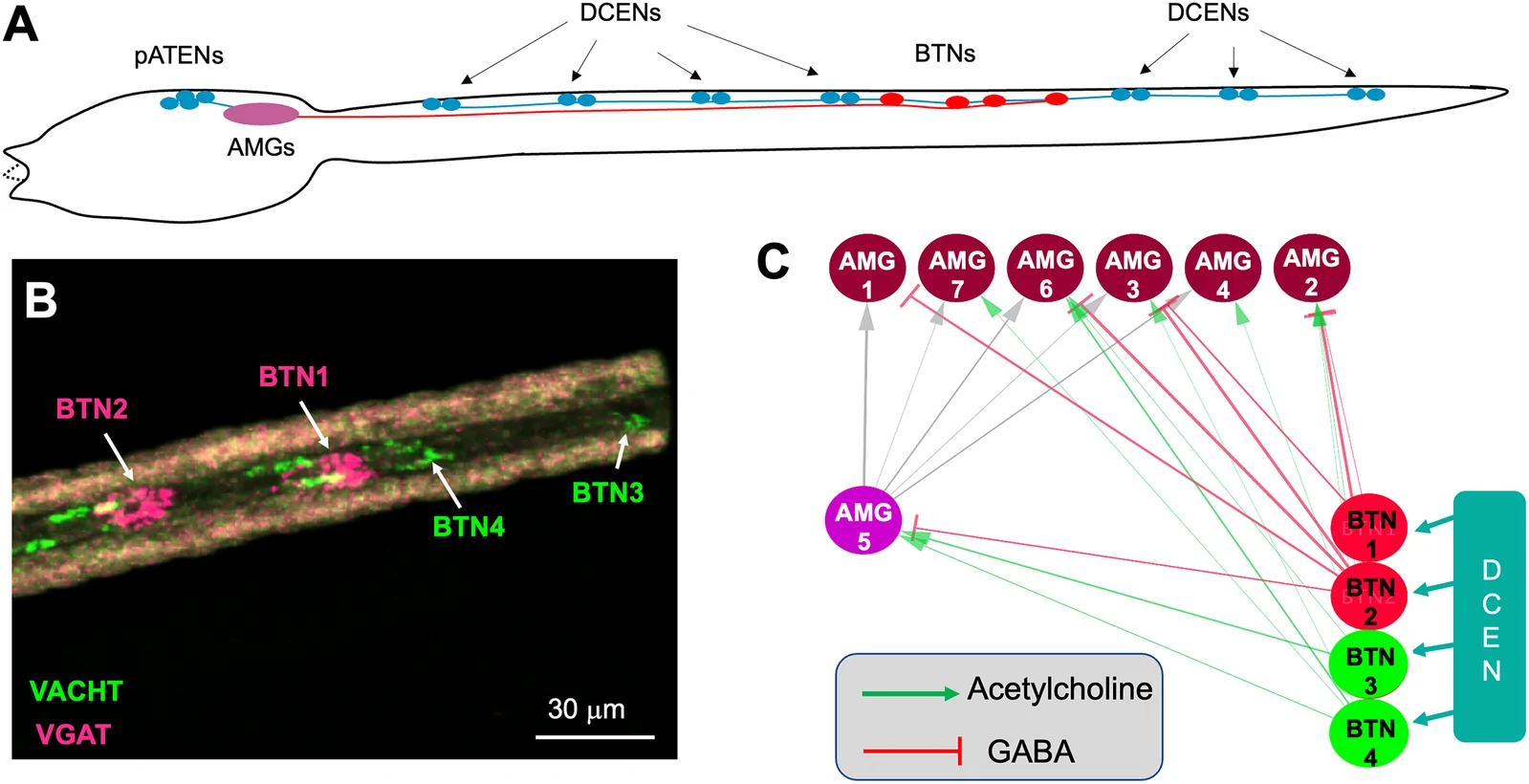
Bipolar tail neurons and the AMG group. ***A***, Relative positions of the pATENs, DCENs, AMGs, and BTNs in a *Ciona* larva [after Ryan et al. (2018)]. ***B***, BTNs labeled by in situ hybridization for VACHT (green) and VGAT (magenta) and outlined (dotted). Smaller, elongate VACHT-expressing cells are tail motor neurons. ***C***, Synaptic input from the BTNs to the AMG group. See Figure 1 legend for abbreviations. See Extended Data Figure 3-1.

In addition to receiving direct mechanosensory input from the pATENs at AMG5, the AMGs are postsynaptic to the four bipolar tail neurons (BTNs), which function as relay interneurons for the mechanosensory dorsal caudal epidermal neurons (DCENs), which are found along the tail (Fig. 3*A*). The BTN neurons are thought to be homologous to neurons of the vertebrate dorsal root ganglia (Stolfi et al., 2015), consistent with their hypothesized role of providing proprioceptive input on tail movements (Torrence and Cloney, 1982; Madden et al., 2020). We find two anterior VGAT-positive (VGAT^+^) BTNs (BTN1 and 2) and two posterior VACHT^+^ BTNs (BTN3 and 4; Fig. 3*B*, Extended Data Fig. 3-1), consistent with previous reports (Kim et al., 2020). Thus, the AMG group appears to receive both positive and negative representations of tail movements. The potential significance of BTN input for the signal processing functions of the AMG group, and how this may tie the AMG group to cerebellum-like structures found in certain vertebrates, is presented in the Discussion.

10.1523/ENEURO.0362-25.2026.f3-1 Figure 3-1 ** ** General view of the larva shown in Figure 3b, highlighting the BTNs. BTN positions are indicated by magenta or green arrows according to their VACHT or VGAT expression and by number (after Ryan et al., 2018). The elongate cholinergic (VACHT positive) tail motor neurons show overlap along the A/P axis with the two anteriormost BTNs. Download Figure 3-1, TIF file.

### Gene expression in the AMG group

**Figure 4.**
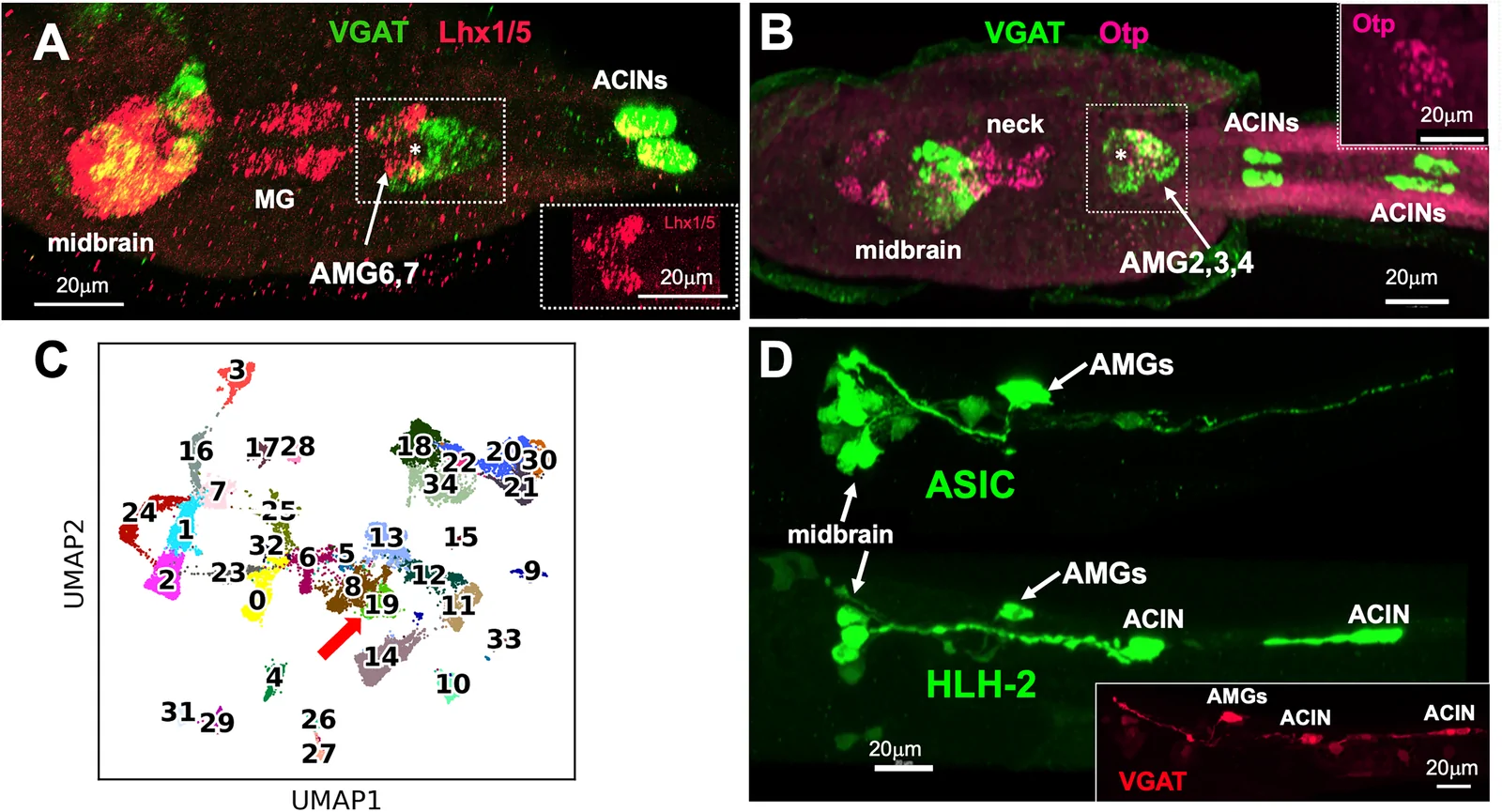
Gene expression in the AMG group. ***A***, ***B***, Expression of Lhx1/5 in a *Ciona* larva by in situ hybridization (panel ***A***, magenta) and Otp (panel ***B***; magenta). Both panels also show expressions of VGAT (green). ***C***, UMAP plot depicting cluster analysis results for single-cell transcriptomes from combined datasets of *Ciona* larvae. The red arrow indicates candidate cluster 19 which corresponds to the putative inhibitory AMGs. ***D***, Larval stage expression of ASIC and NSCL-2 > GFP reporter constructs. ***A***, ***B*** are dorsal views, and ***D*** is lateral. See extended expression data in Extended Data Figures 4-1–4-3. Asterisks in panels ***A*** and ***B*** indicate areas appearing not to express the in situ markers.

The above sections detail that the dorsal and ventral hindbrain of *Ciona* larvae can be distinguished by anatomy, cell-type composition, function, and circuit architecture. These differences will, of course, be reflected in differential gene expression between the two domains. In vertebrates, the dorsal domain is the cerebellum. Recognizing the apparent similarity in the partitioning of the hindbrain in both vertebrates and *Ciona* to dorsal and ventral domains, we started our investigation of the AMG group by examining genes known to be expressed in the cerebellum, starting with the transcription factors Lhx1/5 and Otp, which are expressed in nonoverlapping subpopulations of inhibitory neurons in the cerebellum (Schilling, 2024). We find that in *Ciona* larvae both Lhx1/5 and Otp are expressed in the inhibitory AMGs, with Lhx1/5 expressed in the anterior two AMGs (AMG6 and 7; Fig. 4*A*), and Otp expressed posteriorly in AMGs 2, 3, and 4 (Fig. 4*B*). We also see central domains for both in situ that appear to be free of VGAT and Lhx1/5/Otp expression (Fig. 4*A*,*B*, asterisks). We have previously published that AMG5, which is at the center of the AMG group, does not express VGAT (Kourakis et al., 2019). This indicates that AMG5 also does not express Lhx1/5/Otp. Lhx1/5 and Otp are not confined to AMG neurons but also show expression outside of the AMG cells (Fig. 4*A*,*B*).

10.1523/ENEURO.0362-25.2026.f4-1 Figure 4-1 Phylogenetic analysis of chordate Ptf1a family members. The putative *Ciona* Ptf1a ortholog (yellow highlight) groups with the fer-like class of transcription factors. Despite the absence of a Ptf1a ortholog in *Ciona*, an orthologous gene is present in cephalochordates (Branchiostoma lanceolatum; blue highlight). Download Figure 4-1, TIF file.

10.1523/ENEURO.0362-25.2026.f4-2 Figure 4-2 Heat map of mean cosine similarity between annotated mouse cerebellum single cell RNA sequencing clusters and *Ciona* clusters. Several *Ciona* clusters showed considerable expression similarities with mouse cerebellar clusters. However, mouse Purkinje cells cluster does not show obvious homologous clusters in *Ciona*. Download Figure 4-2, TIF file.

10.1523/ENEURO.0362-25.2026.f4-3 Figure 4-3 Differentially expressed genes in the inhibitory AMG single-cell transcriptome cluster. Differential expression (DE) is listed in descending order of log_2_ mean fold-change (*mean lfc*). DE genes with a *mean lfc* less than 4.8 and an absolute expression level (*raw normalized mean*) less than 0.2 were excluded. Compiled *mean lfc* for all annotated genes in all clusters is available at https://doi.org/10.5281/zenodo.15320250. Genes with no apparent vertebrate homolog as assessed by BLAST analysis (cutoff E< 0.05) are highlighted in orange. Yellow highlighting corresponds to genes that were used to identify the cluster (VGAT, Lhx1/5 and Otp). Select genes with known expression and/or function in the cerebellum are highlighted in green, along with a reference. Translated *Ciona* gene models [KY2021} can be downloaded from http://ghost.zool.kyoto-u.ac.jp/datas/HT.KY21Gene.protein.2.fasta.zip. Download Figure 4-3, TIF file.

To gain a fuller picture of the transcription profile of the AMGs, we took advantage of the availability of two single-cell RNAseq datasets for *Ciona* larvae (Cao et al., 2019; Sharma et al., 2019). As detailed in the Materials and Methods, these two datasets were combined to make a single dataset. Differential gene expression analysis identified a single cluster coexpressing VGAT, Otp, and Lhx1/5 (Fig. 4*C*, cluster 19; Extended Data Fig. 4-3), suggesting that it may correspond to the inhibitory AMGs. The presence of differentially expressed Otp and Lhx1/5 in the same cluster suggests that the Otp^+^ and Lhx1/5^+^ AMGs are very similar. To validate cluster 19 as corresponding to the inhibitory AMGs, we examined the expression of two of its most highly differentially expressed genes: the *Ciona* orthologs of the Acid-sensing ion channel (ASIC; Coric et al., 2008) and Helix-loop-helix protein 2 (HLH-2; Extended Data Fig. 4-3). To assess the expression of ASIC in *Ciona* larvae, we used a previously described expression construct with the *CiASICb* enhancer driving GFP (Coric et al., 2008), while for HLH-2 we made a new construct with ∼1.5 kb 5′ flanking region driving GFP (Materials and Methods). We observed strong expression from both the ASIC and HLH-2 expression constructs in the AMGs, as well as elsewhere in the CNS. We note, however, that the sites of expression outside the AMGs differ between the two constructs, as might be expected for genes identified specifically for their differential expression in the AMGs. The HLH-2 construct also drove expression in the ACINs of the ventral hindbrain, which can be identified by their posterior placement within the ventral hindbrain and their expression of VGAT (Fig. 4*D*, inset). These expression patterns are consistent with cluster 19 corresponding to the inhibitory AMGs. In addition, both ASIC and HLH-2 are differentially expressed in Purkinje (i.e., inhibitory) neurons of the mammalian cerebellum (Extended Data Fig. 4-3; Haire and Chiaramello, 1996; García-Añoveros et al., 1997; Waldmann et al., 1997; Krüger and Braun, 2002). We lack sufficient expression data on AMG5 to perform a similar analysis, other than it expresses VACHT (Kourakis et al., 2019). Moreover, because there is only one AMG5 neuron per larva, single-cell transcriptome analysis may not yield sufficient data to allow for its identification.

### Development of the AMGs

**Figure 5.**
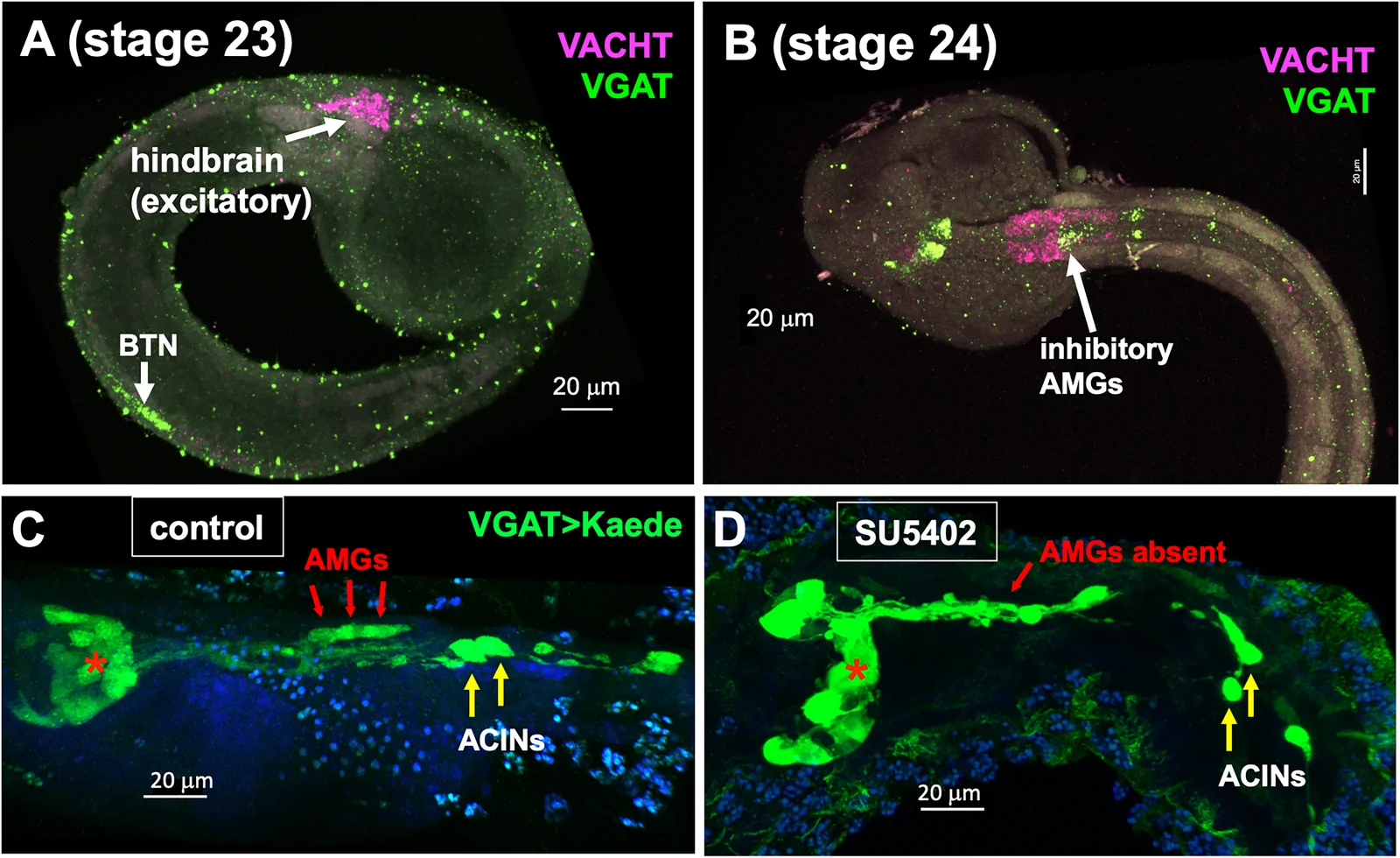
Development of the AMG group. ***A***, ***B***, Expression of VGAT (green) and VACHT (magenta) in the developing hindbrain. Anterior is to the right. ***C***, Vehicle control for SU5402 experiment (DMSO treatment). Larvae expressing Kaede fluorescent protein driven by the VGAT promoter and labeled with anti-Kaede primary antibody and an Alexa Fluor secondary (red arrows). ***D***, SU5402 treatment eliminates the VGAT-expressing AMGs. The presence or absence of AMG5, however, which would not be labeled by the VGAT reporter, cannot be directly determined in our assay. Red asterisks in panels ***A*** and ***B*** indicate midbrain VGAT^+^ neurons. BTN, bipolar tail neurons; AMG, ascending motor ganglion interneurons, ACIN, ascending contralateral inhibitory neurons. In ***C*** and ***D*** anterior is to the left.

We investigated the timing of the inhibitory AMG neuron development by examining the onset of expression of VGAT, determined by in situ hybridization, in order to compare their induction to the dorsal hindbrain of vertebrates. At mid-tailbud stages (Stage 23), we observed no VGAT expression in the developing hindbrain, although expression was detected in the developing inhibitory BTNs of the tail. However, at this stage VACHT was detected in the hindbrain (Fig. 5*A*). Because there is only one VACHT^+^ neuron in the dorsal hindbrain, most, or all, of the VACHT expression at this stage is in the ventral hindbrain. By Stage 24, VGAT expression was detected in the dorsal hindbrain (Fig. 5*B*). We observed this pattern of expression, with the VACHT^+^ hindbrain neurons developing before the VGAT^+^ neurons, in each of four samples examined. The timing of these expression patterns, including the earlier detectable VGAT in the caudally positioned BTNs, as well as the timing of VGAT/VACHT in the midbrain, could give clues as to how functional circuits for sensorimotor pathways are established temporally. The published connectome is for a *Ciona* larva; earlier stage-specific connectomic data could provide insight into the sequential development of neural circuits.

In vertebrates, the dorsal hindbrain is induced via the action of FGF8 produced at the MHB (Butts et al., 2014). The *Ciona* ortholog of this gene, called FGF8/17/18, shows a remarkably well-conserved expression pattern in the developing CNS, being expressed in the CNS neck region, immediately posterior to neurons expressing *OTX* and *engrailed* (*En*), as in vertebrates (Imai et al., 2002). The naming of FGF8/17/18 reflects the multiple vertebrate orthologs of the single *Ciona* gene, resulting from independent whole-genome duplications that occurred in the vertebrate lineage, after vertebrate and tunicate lineages diverged (Dehal and Boore, 2005). Based on morphology, gene expression, and inductive activity, the *Ciona* larval CNS neck region has been equated with the MHB (Wada et al., 1998; Ikuta and Saiga, 2007). Previous studies showed that the loss of FGF8/17/18 disrupts motor ganglion gene expression (Imai et al., 2009), although disruptions specific to the AMG group were not addressed. To investigate a possible role for FGF in the development of the AMG group, we treated *Ciona* embryos with the FGF receptor inhibitor SU5402. To assess the presence/absence of the AMG group, we made use of a stable transgenic line expressing Kaede fluorescent protein under the control of the VGAT promoter (Horie et al., 2011), which labels the six inhibitory AMG neurons (1–4 and 6–7). We found that treatment of the embryos for 30 min with 20 µmol SU5402 at the late neurula stage eliminated VGAT expression in the dorsal hindbrain, while a vehicle control (DMSO) had no effect (Fig. 5*C*,*D*). Despite the loss of the AMGs, other VGAT-expressing CNS neurons appeared to be present, including those in the midbrain (red asterisks) and the ACINs of the motor ganglion, indicating that the AMGs are uniquely induced at this developmental stage. Treatment of older embryos with SU5402, from the initial tailbud stage onward, had no effect on the development of the AMG group (data not shown).

Because *Ciona* has a single FGF receptor, all *Ciona* FGF members will be inhibited when SU5402 is present, not just FGF8/17/18 (moreover, the mechanism of SU5402, while specific to FGF receptors, is not specific to FGF receptor subtypes). In addition to FGF8/17/18, other FGF members known to be expressed during embryogenesis include FGF3/7/10/22, FGF9/16/20, FGF11/12/13/14, and FGFL. Of these, two candidates show similarities to FGF8/17/18 expression: FGF3/7/10/22 and FGF9/16/20. FGF3/7/10/22 is expressed in tailbud embryos in the ventral nerve cord overlying the notochord, extending from the neck region to the tip of the tail; it has a demonstrated role in convergent extension, the movements characterizing the coming into alignment of notochord cells to the midline, in single file (Shi et al., 2009). FGF9/10/16 is expressed in the posterior sensory vesicle/midbrain, anterior to the neck, where FGF8/17/18 is expressed (Imai et al., 2002). The orthology of FGF8/17/18 with vertebrate FGF8, as well as the putative homology of the neck region to the MHB organizing region, suggests that the *Ciona* ortholog to FGF8 is most likely to be involved during induction of the dorsal aspect of the motor ganglion. Consistent with this, a previous study showed that knockdown of FGF8/17/18 with a morpholino resulted in expansion of markers in the pSV (midbrain homolog) of tailbud embryos (Imai et al., 2009). The authors draw parallels to the zebrafish ace (Fgf8a) mutant and conditional mouse mutants showing similar transformations.

### Inhibitory AMGs have spontaneous spiking activity

**Figure 6.**
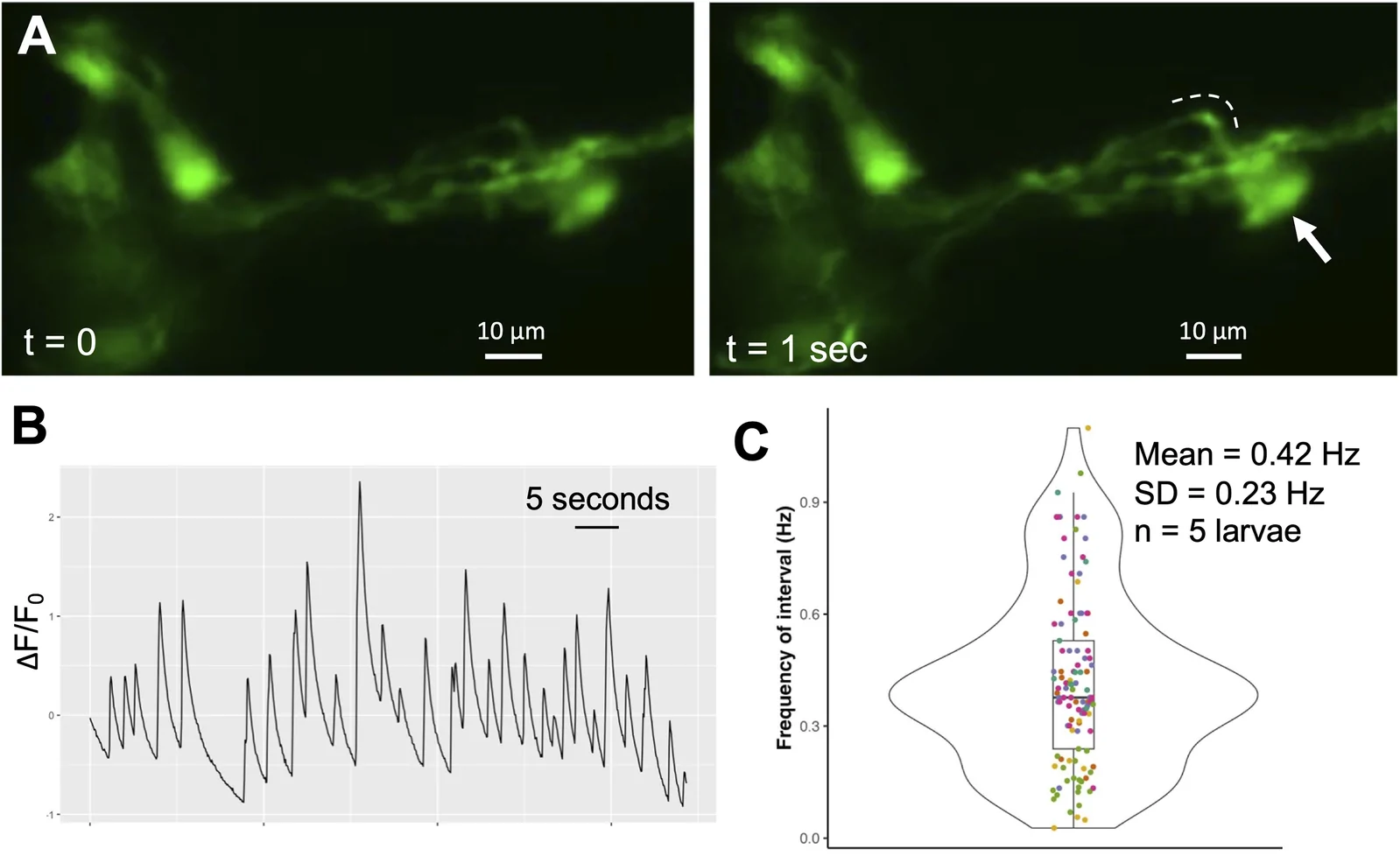
Spontaneous activity in the inhibitory AMGs. ***A***, Representative example of a calcium transient in the AMG group detected with GCaMP6f driven by the VGAT promoter. The calcium transient is observed at *t* = 1 s as increased fluorescence in the AMG group (arrow) and in an ascending axon (dashed arrow). Images are frames taken from Movie 2. Anterior is to the right. ***B***, Representative plot of a GCaMP6f fluorescence in the inhibitory AMG group. ***C***, Combined data on spontaneous activity in the AMG group from five larvae. Data from each larva are a different color, and dots correspond to all 1/peak interval times in seconds for each recording and were used to determine mean frequency in Hz. The box indicates the median values (middle bar) and first to third interquartile ranges (top and bottom bars); whiskers indicate 1.5× the interquartile ranges. See Extended Data Figure 6-1 and Movie 2.

Given the similar anatomical location, gene expression, circuit architecture, and development of the AMG group and neurons of the vertebrate dorsal hindbrain, we extended our analysis to determine whether the inhibitory AMG neurons might display spontaneous spiking activity, as has been observed in some inhibitory neurons of the cerebellum (Purkinje cells). In vertebrates, this spontaneous activity consists of both high-frequency “simple spikes” (>40 Hz), and trains of slower (1–3 Hz) “complex spikes” (Raman and Bean, 1999; Streng et al., 2018; Otis, 2023). In the present experiment, we electroporated one-cell stage *Ciona* embryos with a plasmid containing GCaMP6f driven by the VGAT promoter (VGAT > GCaMP6f) and then assessed animals at the larval stage (Chung et al., 2023). The temporal resolving power of calcium transients by GCaMP is below what is needed to detect simple spikes, but we have successfully used GCaMP to quantify rhythmic firing behavior of other neurons in *Ciona* larvae on the time scale of complex spikes (Chung et al., 2023). Figure 6*A* shows two frames from a representative recording of GCaMP activity in which a spike (calcium transient) was observed in the AMG group (Movie 2, arrows). Five GCaMP-expressing larvae were imaged, and Figure 6*B* shows a representative plot of normalized fluorescence (Δ*F*/*F*_0_) versus time. In the five larvae analyzed (Extended Data Fig. 6-1), we observed trains of calcium transients, sometimes persisting through the imaging sessions (∼3 min), but in other cases occurring in bursts lasting tens of seconds (Fig. 1, larva 4). In analyzing fluorescence data, we selected small regions of interest (ROIs) for quantification that correspond to the approximate size of individual cells. While it is possible these ROIs contained more than one cell, we also observed temporally matched calcium transients in separate AMGs (Fig. 1, larva 1), indicating that they are coupled. The mean spiking frequency for the inhibitory AMG neurons from five larvae was 0.42 ± 0.23 Hz (Fig. 6*C*). Overall, the pattern of calcium transients in the inhibitory AMGs appears similar to complex spikes (Davie et al., 2008), although their frequency is lower. The lower frequency may indicate different mechanisms between the inhibitory AMGs and the Purkinje cells, and these spontaneous activities may be unrelated. However, it is also well documented that the frequency of rhythmically activity neurons is temperature dependent (Tang et al., 2010), and *Ciona* were imaged at 18°C.

10.1523/ENEURO.0362-25.2026.f6-1 Figure 6-1 Spontaneous spiking activity in the inhibitory AMG neurons. Data from five larvae transfected with the plasmid VGAT>GCaMP6f. Arrows in left panels indicate the neuron(s) analyzed. Right panels show plots of normalized GCaMP6f fluorescence (DF/F_0_). Two regions of interest (ROI), corresponding to separate neurons, were analyzed for larva 1. In all other larvae, a single ROI was analyzed (arrows). Download Figure 6-1, TIF file.

**Movie 2.** Spiking activity of AMGs. Calcium transients in a *Ciona* larva expressing VGAT > jGCaMP6f. The movie shows 47 s of recording at 8.7 frames/s. The movie plays at 5× speed. [View online]

## Discussion

### The *Ciona* larval hindbrain

Evidence for CNS homology between long diverged chordate species takes many forms, including shared anatomy, development, function, neural circuitry, and gene expression. The examination of homologous characteristics helps to delineate the scope and pace of morphological change over time and to identify the origins of morphological novelty within our own evolutionary lineage. As detailed in the Introduction, multiple lines of evidence support the homology of the *Ciona* larval MG with the vertebrate hindbrain. These include its anatomical location in the *Ciona* larva between the MHB and the spinal cord, shared gene expression, constituent neuron type, and synaptic connectivity (Hudson, 2016; Ryan et al., 2017; Kourakis et al., 2021, 2025).

Nevertheless, in *Ciona* the developmental expression of several genes appear to be confounding—such as the absence of a Gbx ortholog and the atypical expression of Hox10 within the MG (Ikuta et al., 2010). In both vertebrates and cephalochordates (e.g., amphioxus), the outgroup for tunicates and vertebrates (Delsuc et al., 2006), Hox10 paralogues are expressed in the caudal CNS (e.g., lumbar spinal cord for vertebrates; Wu et al., 2008; Pascual-Anaya et al., 2012). Thus, it is most likely that *Ciona* Hox10 expression in the hindbrain is derived within the chordates, consistent with the overall disruption, dispersal, and modification of the *Ciona* Hox cluster (Ikuta et al., 2004, 2010). However, in light of the multiple lines of evidence supporting the homology of the MG and the hindbrain, these differences likely point to plasticity and drift of developmental mechanisms, reflecting changes which have accrued separately in the lineages leading to *Ciona* and to vertebrates, rather than to independent origins of the MG and the hindbrain. A similar mix of conservation and divergence is observed, for example, in comparing gene regulatory networks between tunicate and vertebrate notochords (Kugler et al., 2011; Di Gregorio, 2020). Moreover, such drift may be expected given the large evolutionary distance between tunicates and vertebrates, and the many apparent gene losses and rearrangements in the *Ciona* genome when compared with those of other chordates (Holland and Gibson-Brown, 2003; Ikuta et al., 2004; Simakov et al., 2022). In addition, given the conserved gene expression, morphology, function, and neuron composition of the CNC to the vertebrate spinal cord (Gionti et al., 1998; Takatori et al., 2002; Kourakis et al., 2025), there appears to be little support for the earlier hypothesis that the MG has homology to both the vertebrate hindbrain and spinal cord (Dufour et al., 2006). Nevertheless, the *Ciona* hindbrain is a vastly simpler structure than its vertebrate counterpart, having only thirty neurons (Fig. 1*B*). Accordingly, the *Ciona* larval hindbrain lacks evident subdivisions ventrally, such as pons and medulla oblongata, as found in vertebrates. However, one subdivision that the *Ciona* hindbrain does have is in the dorsal/ventral axis. In *Ciona*, the dorsal and ventral domains differ in function, gene expression, neuron type, and synaptic connectivity. Moreover, it is thought that the dorsal and ventral hindbrains of the *Ciona* larvae develop from different embryonic lineages (Popsuj and Stolfi, 2021).

The gene regulatory pathways of motor- and interneuron specification in the ventral *Ciona* larval hindbrain have been extensively characterized (Stolfi and Levine, 2011; Piekarz and Stolfi, 2024; Kim et al., 2025). In the dorsal hindbrain, the specification of AMG5 (the sole cholinergic AMG) requires the transcription factor *Ebf* (Popsuj and Stolfi, 2021). We show here that the GABAergic AMG neurons are transcriptionally heterogeneous, with the anterior two (AMG6 and 7) expressing the transcription factor Lhx1/5, the middle three (AMG2,3,4) expressing the transcription factor *Otp*, while the most posterior (AMG1) expresses neither of these genes. Whether this heterogeneity of gene expression in the GABAergic AMGs reflects functional differences is unknown, but at the level of synaptic connectivity (Figs. 2*B*, 3*C*) they appear to be equivalent. It is also unknown if either or both Otp and Lhx1/5 are necessary for inhibitory AMG development. Our identification of a scRNA-seq cluster for the inhibitory AMGs provides a tool for future investigation of AMG development and function.

### Did dorsal/ventral subdivision of the hindbrain arise independently in vertebrates and tunicates?

Anatomically, the AMG group occupies an equivalent position in the CNS to the cerebellum of vertebrates (i.e., dorsal hindbrain). However, the simple structure of the AMG, and its function as a relay center for mechanosensory input, clearly distinguishes it from a cerebellum. Moreover, the cerebellum is thought to have emerged following the split of jawed vertebrates (gnathostomes) from the jawless vertebrates (agnathans; Pose-Méndez et al., 2016; Hibi et al., 2017; Lamanna et al., 2023). Nevertheless, embryos of lamprey and hagfish (both agnathans) express the genes *Ptf1a* and *Atonal* in the developing dorsal hindbrain (rhombomere 1; Sugahara et al., 2021). Both of these genes are commonly used markers of GABAergic and glutamatergic neurons on the cerebellum, respectively, although they both are widely expressed elsewhere in the CNS. While the dorsal rhombomere 1 derivatives appear to degenerate in hagfish, the Ptf1a positive cells of lamprey appear to develop into inhibitory neurons, paralleling certain aspects of cerebellum development. However, the dorsal hindbrain domain of lamprey has been described as comprising an undifferentiated plate-like cerebellum, lacking the laminar organization of vertebrate cerebella (Sugahara et al., 2017). This would appear to agree with RNA sequence analysis of the lamprey CNS. One report which made use of scRNA-seq found that while many lamprey neuron types could be linked to mouse neuron types based on transcriptomics, no correspondence was found between lamprey neurons and cerebellar neurons (Lamanna et al., 2023). In a separate study, which combined snRNA-seq with Stereo-seq, the putative lamprey cerebellum-like region was found to have interneurons with a transcription profile suggesting they could represent evolutionary precursors to Purkinje cells, but which crucially lacked expression of canonical Purkinje cell markers (Wu et al., 2026). Thus, while agnathans possess a distinct dorsal hindbrain domain which express molecular markers associated with the cerebellum, this domain may lack other fully developed, cerebellum-defining characteristics. A connectome for lamprey or hagfish, though currently lacking, could supplement expression and RNAseq data, revealing whether there exist commonalities in circuit logic between dorsal hindbrains of cyclostomes and vertebrates. Despite this, conserved gene expression indicates that the presence of distinct dorsal and ventral hindbrain domains predates the gnathostome/agnathan split, with the dorsal hindbrain of the common gnathostome/agnathan ancestor serving as a possible precursor to the cerebellum.

We hypothesize, based on data presented here, that the appearance of dorsal and ventral hindbrain domains is even more ancient, predating even the vertebrate/tunicate split. The alternative that the *Ciona* dorsal hindbrain domain (i.e., AMG group) evolved independently from those of vertebrates appears less plausible given the weight of evidence. In such a scenario, shared patterning and common developmental mechanisms within an anatomically homologous CNS would presumably set the stage for the independent (and parallel) elaboration of the dorsal hindbrain in each lineage. In the scenario we favor, however, the common ancestor already had a dorsal/ventral-regionalized hindbrain; after long and separate evolutionary trajectories, the dorsal domain gave rise to the cerebellum in the lineage leading to vertebrates and the AMG complex in the tunicates. We find conserved gene expression between the AMG group and the cerebellum. While there are no known genes that are exclusive to the cerebellum, we present here are a set of genes differentially expressed in both, including Lhx1/5, Otp, ASIC, VGAT, and HLH-2 (Fig. 4). In addition, cluster 19 contains a number of other genes that are known to be differentially expressed in cerebellum (Extended Data Fig. 4-3, green highlights). *Ciona* Atonal appears to be absent from AMG cells; its expression has been shown in epidermal hair-cell precursors of the tailbud stage embryos (Tang et al., 2013). Another transcription factor that is conspicuously absent in the AMG group is Ptf1a, which is a key regulator of GABAergic neurons in the cerebellum and elsewhere (Butts et al., 2014; Jin and Xiang, 2019). While *Ciona* was purported to have a Ptf1a ortholog (Razy-Krajka et al., 2012), further analysis showed that the gene annotated as *Ciona* Ptf1a is a paralog, not an ortholog, of mammalian Ptf1a and lies within the Fer cluster of the larger Ptf1a-related gene family (Oonuma and Kusakabe, 2021). However, the cephalochordate *Branchiostoma lanceolatum* does appear to have a true Ptf1a ortholog (Extended Data Fig. 4-1). Because the cephalochordate subphylum of the chordates is basal to both the tunicates and vertebrates (Delsuc et al., 2006), it appears most likely that the tunicates lost the Ptf1a gene. Whether cephalochordates have a distinct dorsal hindbrain domain, and whether it expresses Ptf1a, has not been explored.

Finally, we compared the single combined *Ciona* brain transcriptome to a mouse cerebellum transcriptome, but no convincing cellular homologies between the two species were observed (data not shown). While this is a negative result, this may be a reflection of the evolutionary distance between tunicates and vertebrates . We performed a comparison of the transcriptomes of all *Ciona* larval clusters shown in Figure 4*C* to a set of mouse cerebellum scRNA-seq transcriptomes (Kozareva et al., 2021). Briefly, we identified one-to-one orthologs between the mouse and *Ciona* genomes, used these orthologs to map mouse gene expression to corresponding *Ciona* genes, and measured the cosine similarity between mouse and *Ciona* clusters (see Materials and Methods). While there were varying degrees of match between human and *Ciona* single-cell transcriptomes, none of the *Ciona* clusters was a good match for the predominant cell types in the core circuit of the cerebellum (Purkinje and granule; Extended Data Fig. 4-2). While this result appears similar to lamprey, the enormous evolutionary distance between tunicates and vertebrates, as well as the much greater neuron-type diversity of vertebrates compared with tunicates, make this approach of questionable validity.

We conclude that the gene expression data presented here is consistent with the hypothesis that the AMG group has a common origin with the dorsal hindbrains of vertebrates. However, the absence of definitive markers for the vertebrate dorsal hindbrain prevents a stronger conclusion based on gene expression alone.

### Is the AMG group a cerebellum-like structure?

The *Ciona* larva has a complex peripheral nervous system that projects to the CNS at several relay centers, the AMG group being one of them (Ryan et al., 2018). However, the circuitry of the AMG group stands apart and suggests a circuit architecture similar to those found in the vertebrate cerebellum-like structures, although on a vastly simplified scale, and with some notable differences. Vertebrate cerebellum-like structures are anatomically and functionally distinct from the cerebellum and receive, process, and relay direct sensory inputs from both mechano- and electrosensory neurons (Bell, 2002; Bell et al., 2008; Montgomery et al., 2012; Sawtell and Bell, 2013). Examples include a cerebellum-like structure found in the dorsal cochlear nucleus of mammals that receives and processes mechanosensory input from the auditory nerve and a cerebellum-like structure found in the medial octavolateral nucleus of fish and amphibians that receives input from the mechanosensory lateral line (Singla et al., 2017; Perks et al., 2020). The core neural circuits of the cerebellum and cerebellum-like structures are similar, with excitatory granule cells (or granule-like cells in cerebellum-like structures) branching to a network of parallel fibers making multiple synaptic contacts with inhibitory Purkinje/Purkinje-like cells. In the *Ciona* AMG group, inputs from the mechanosensory pATENs project to the central cholinergic neuron, AMG5 (Fig. 2*B*). AMG5, with its large bifurcating axon, then branches to form synapses with the inhibitory AMGs (Fig. 2*B*,*C*). Also as in cerebella and cerebellum-like structures, the output is inhibitory (e.g., Purkinje cells in the cerebellum and the inhibitory AMGs in *Ciona*). Thus overall, the circuit architecture of the AMG group with a branching excitatory input neuron synapsing to multiple inhibitory output neurons appears similar to cerebellum and cerebellum-like structures, although functionally the AMG group appears to have more in common with cerebellum-like structures. In this circuit, the inhibitory AMG neurons would be functionally similar to Purkinje cells, while AMG5 would have a function akin to granule cells. Despite this similarity, important differences are evident as well; the most obvious being that in cerebellum and cerebellum-like structures the core processing circuit is repeated on a numerically vast scale, while in the AMG group there is only a single circuit consisting of a single excitatory input neuron and six inhibitory output neurons. However, it should be noted that the small number of neurons in the AMG group is in keeping with the overall small number of neurons in all classes in *Ciona* larvae (Ryan and Meinertzhagen, 2019), and is perhaps a reflection of a simplification of tunicates from a more complex ancestor (Holland, 2016).

A second noticeable difference between the AMG group and cerebellum/cerebellum-like structures is that the excitatory input to each makes use of different neurotransmitters: AMG5 is cholinergic while granule cells are glutamatergic. While we do not have evidence, nor do we claim, that AMG5 and granule cells themselves share a single origin indicating a direct one-to-one homology, it is well documented that homologous neurons can switch neurotransmitter use (Tosches, 2017). It is also worth noting that *Ciona* larvae are overall lacking glutamatergic interneurons; the only glutamatergic neurons in *Ciona* larvae are sensory (photoreceptors, antennae cells, and peripheral neurons; Kourakis et al., 2019). However, glutamatergic interneurons are present in cephalochordates (amphioxus), suggesting that the lack of glutamatergic interneurons may be a derived feature of tunicates (Candiani et al., 2012) and that for unknown reasons, tunicates have either lost glutamatergic interneurons or changed their neurotransmitter use. This change from glutamatergic to cholinergic may also be evident in the putative *Ciona* reticulospinal neurons (Ryan et al., 2017; Kourakis et al., 2025), which are cholinergic, rather than glutamatergic as in vertebrates (Kourakis et al., 2019; Chopek et al., 2021).

Functionally, cerebellum-like structures not only receive sensory input but also act as predictive cancellation filters (Bratby et al., 2017; Montgomery and Perks, 2019; Perks et al., 2020). Cancellation filters are essential for countering sensory input, known as reafference, that arise from self-stimulation of mechanosensory neurons by the animal's own motor behavior. The cerebellum-like structures receive corollary discharge and proprioceptive inputs which are then used to predict or subtract self-generated signals (Crapse and Sommer, 2008; Requarth et al., 2014; Straka et al., 2018). It is likely that *Ciona* larvae would face the same self-stimulation problem as observed in other animals, both invertebrate and vertebrate (Jékely et al., 2021). Examination of the *Ciona* connectome dataset reveals a plausible predictive cancellation filter functionality in the AMG group. The AMGs, in addition to receiving direct mechanosensory input from the pATENs at AMG5, are postsynaptic to the four bipolar tail neurons (BTNs; Fig. 3*C*). The BTNs serve as relay interneurons for the mechanosensory dorsal caudal epidermal neurons (DCENs; Ryan et al., 2018), which have a proposed proprioceptive function (Torrence and Cloney, 1982). Significantly, the BTNs have mixed valence, with the anterior two (BTN1 and 2) being VGAT^+^ (i.e., inhibitory), while the posterior two (BTN3 and 4) are VACHT^+^ (i.e., excitatory; Fig. 3*B*). The output from the BTNs should thus provide both positive and negative representations of tail movements to the AMG group, as is seen in cerebellum-like structures. Whether the BTN input to the AMGs functions in reafference cancellation remains to be determined.

In summary, we present here a new perspective on the evolution of the cerebellum: the *Ciona* larval CNS not only possesses a domain that shares homology to the vertebrate hindbrain (as we outline in the Introduction), but we further specify in this communication that this homology includes functionally distinct dorsal and ventral domains. The features tying the AMG group to the vertebrate dorsal hindbrain are numerous, and include gene expression, developmental induction, circuit architecture, and spiking activity. Taken as a whole, these data suggest that the cerebellum evolved from a structure that was already present before the tunicate/vertebrate split. Moreover, it has been proposed that the cerebellum evolved from a cerebellum-like structure (Bell, 2002; Montgomery et al., 2012), suggesting that the cerebellum evolved from a structure that was already partially prefigured. Connectome data on the hindbrains of both lamprey and cephalochordates would likely be informative. In lamprey, we predict that the circuit architecture of the dorsal hindbrain would resemble a cerebellum-like structure. Connectome data from cephalochordates could indicate if the dorsal and ventral hindbrain structures were present prior to the split of the cephalochordates from the olfactors (tunicates and vertebrates).

## Synthesis

Reviewing Editor: Charlotte Cornil, Universite de Liege Decisions are customarily a result of the Reviewing Editor and the peer reviewers coming together and discussing their recommendations until a consensus is reached. When revisions are invited, a fact-based synthesis statement explaining their decision and outlining what is needed to prepare a revision will be listed below. The following reviewer(s) agreed to reveal their identity: NONE.

Review Summary

This manuscript has now been examined by two experts who both find the question of interest and agree on the overall quality of the methodological approaches and observations. However, they find the presented evidence insufficient to conclude that the AMG is homologous to, or a precursor of, the vertebrate cerebellum and as a result consider that the conclusions are overstated. They also note the novelty of some observations or approaches/tools is at times difficult to assess. Therefore, our main recommendations are to interpret the observations more neutrally, tone down the conclusions and make more explicit where findings or tools are novel in the text. More detailed assessments and specific recommendations and suggestions are provided here below.

Detailed assessments and specific comments

This study investigates the central nervous system of the ascidian Ciona larva, focusing on the motor ganglion (MG) located posterior to the Neck region. The authors propose that the MG is divided into two functional domains along the dorsoventral axis and that the dorsal ascending motor ganglion interneurons (AMGs) may represent an ancestral form of the vertebrate cerebellum or a cerebellum-like structure. They used state-of-the-art tools to examine this hypothesis from multiple perspectives, comprehensively describe and functionally interrogate the connectome, gene expression, development and neurophysiological characteristics of the AMG neurons of the Ciona larva.

The observations and analyses of the AMGs are carefully performed, and the identification of distinct neuronal circuits and functions along the dorsoventral axis of the MG provides valuable insights indicating that the MG region posterior to the Neck in Ciona exhibits dorsoventral circuit differences, and that the dorsal AMG neurons function as a relay center for sensory input. The scRNA-seq analysis elucidating the AMG gene profile is also valuable. Nevertheless, the hypothesis that Ciona AMGs (a group of neurons) and the vertebrate cerebellum (a brain structure) share a common evolutionary origin-that is, true homology-remains inadequately supported. The developmental and genetic mechanisms differ substantially, making it difficult to view the AMG group as a cerebellar precursor. It is equally plausible that the AMG circuit represents a case of convergent evolution, in which a similar circuit motif was independently acquired. Because alternative explanations were less considered, the conclusions of this manuscript appear overstated. Moreover, what aspect of the findings or what tools/approaches are novel it is not always clear.

Therefore our main recommendations are to interpret the observations more neutrally and tone down the conclusions as well as make more explicit where findings or tools are novel in the text. Rather than emphasizing fragmentary similarities with the cerebellum, we believe that the study would be stronger if presented as a detailed description of the neural circuitry and gene expression patterns of the dorsal AMG neurons in Ciona rather than as evidence for cerebellar homology. Specific comments are listed here below and organized based on their nature and the importance.

Specific comments

Major issues relative to the conclusion

- Neural Circuitry - The authors report that the "hindbrain" region posterior to the Neck contains distinct dorsal and ventral neural circuits and that the dorsal AMG functions as a relay center transmitting sensory information anteriorly. This finding is informative and contributes to our understanding of ascidian neurocircuitry. However, dorsoventral differences in neuronal distribution have already been reported in other regions of the Ciona CNS (Ryan et al., 2016, eLife), and such differences alone do not justify interpreting the dorsal region as a cerebellar prototype. Moreover, as the authors themselves acknowledge, the AMG circuitry differs in many respects from that of the cerebellum and more closely resembles that of a "cerebellum-like structure." The idea that the cerebellum evolved from a cerebellum-like structure remains hypothetical and is not yet supported by robust evidence.

- Interpretation of Developmental Position and Circuit Homology - The authors define the region anterior to the Neck (a constriction in the neural tube) as the midbrain and the posterior region as the hindbrain, and depict this scheme in their figures. This interpretation remains speculative and may mislead readers. As noted in the Introduction, homology of the midbrain and hindbrain between ascidians and vertebrates is only weakly supported by gene-expression patterns and relies primarily on functional correspondence inferred from connectomic data. The present study assumes the correctness of this pre-existing connectomic hypothesis and then further extends it to claim cerebellar homology. However, it is logically problematic to build a higher-level homology (with the cerebellum) upon an unverified lower-level homology (midbrain-hindbrain identity). While the Neck region indeed exhibits gene-expression similarities to the vertebrate midbrain-hindbrain boundary (MHB), MHB-like domains are also found in hemichordates lacking a central nervous system (Pani et al., 2012, Nature). Thus, MHB-like expression cannot by itself define adjacent midbrain and hindbrain regions. Furthermore, as the authors acknowledge, the key transcription factor Gbx, essential for hindbrain and cerebellar development in vertebrates, is absent from the ascidian genome.

- Ptf1a - The transcription factor Ptf1a, indispensable for Purkinje cell differentiation in the vertebrate cerebellum, is not expressed in the Ciona AMG. The authors' phylogenetic analysis (Fig. 4-1) argues that the known Ciona "Ptf1a" gene is actually an ortholog of the vertebrate Fer-3-like gene and that the true Ptf1a ortholog was lost in the tunicate lineage. However, the possibility that Ptf1a and Fer3-like genes diverged following the two rounds of whole-genome duplication (2R-WGD) in the vertebrate ancestor is not discussed. It is plausible that the ancestral Ptf1a/Fer3-like gene (including twist-like gene?) existed as a single copy in amphioxus and tunicates and that vertebrate Ptf1a and Fer3-like genes arose via duplication during 2R-WGD. If so, the Ciona "Ptf1a," even if clustered within the Fer3-like branch, could still represent the orthologous counterpart of vertebrate Ptf1a. Notably, the amphioxus Fer3-like gene was not included in the tree. The authors should re-analyze and clarify this issue.

- Excitatory Neuron Differentiation and Atoh1 - Genes essential for the differentiation of excitatory granule cells in the vertebrate cerebellum, such as Atoh1, are not discussed. Although the authors show that AMG5 is excitatory, they acknowledge that it is cholinergic rather than glutamatergic, differing from cerebellar granule cells. They attribute this to a derived neurotransmitter change in tunicates, but if this is the case, additional molecular evidence-such as the expression of Atoh1-is required to support any proposed homology between AMG5 and granule cells. Without such evidence, it is difficult to regard AMG5 as sharing a common origin with cerebellar granule cells.

- Gene-Expression Analysis - Using scRNA-seq and in situ hybridization, the authors identify the gene profile of the inhibitory AMG neurons, reporting expression of Lhx1/5, Otp, ASIC, VGAT, and HLH-2. This comprehensive analysis is valuable; however, none of these genes is specific to the cerebellum-they are broadly expressed in other CNS regions. For example, Lhx1/5 is expressed in dorsal inhibitory interneurons of the spinal cord (Pillai et al., 2007, Development), and Otp is expressed in the hypothalamus (The Mouse Nervous System, 2012) and in inhibitory interneurons of the spinal cord (Worthy et al., 2024, eLife). Moreover, the vertebrate cerebellum arises exclusively from rhombomere 1, a segment that lacks expression of any Hox genes (Butts et al., 2014, Development). In contrast, Ciona AMGs express Hox10, and the authors even use the Hox10 cis-regulatory region to visualize AMG cells. While they argue that Hox10 expression represents a derived tunicate feature, their selective emphasis on genes that support cerebellar similarity (while attributing discordant patterns, such as Ptf1a absence or Hox10 expression, to lineage-specific modification) undermines the objectivity of the discussion. A more systematic, unbiased method of cell type comparison between Ciona and other non-jawed and jawed vertebrate species would be necessary to solve the homology question - if at all it can be solved, given the small number of neurons.

- FGF-Signaling Inhibition Experiments - The authors treat embryos with the FGF-receptor inhibitor SU5402 and report loss of AMG cells, suggesting that AMG development depends on FGF signaling as in the vertebrate cerebellum. The SU5402 treatment effect in panels C-D of Figure 5 are a bit difficult to compare. How can we be sure the treatment did not affect the placement of the neurons? Adding a more specific marker for AMG would more convincingly show absence of these neurons. Moreover, FGF signaling is essential in many brain regions, including the anterior neural ridge (ANR) of the telencephalon. Thus, FGF dependence alone is merely circumstantial evidence and cannot demonstrate developmental homology. Furthermore, in Fig. 5D, not only the AMG domain but also adjacent VGAT-positive neurons exhibit marked morphological alterations. This raises the possibility that the observed effects reflect generalized developmental delay or nonspecific morphological defects in the CNS. Consequently, concluding that AMG development shares the same mechanism as cerebellar development seems overstated. In addition, SU5402 inhibits multiple receptor tyrosine kinases and is not specific to the FGF8 pathway.

- Spontaneous spiking is a feature of many neurons, also neurons outside of the cerebellum. It is not entirely clear how the measurements shown in Figure 6 contribute to the research question of cellular homology with cerebellar cells. The evidence that is presented here seems too preliminary to draw big conclusions from, and other types of analysis that can distinguish individual cells could bring deeper insight into firing characteristics, also upon stimulation.

- The question whether dorsal hindbrain of Ciona (AMG cells) are evolutionary homologs of cerebellum in vertebrates has not been completely addressed, and while there are specific aspects that hint towards this relationship, these were not complete enough to be fully convincing. A more thorough investigation of the cell type homologies is missing, and other assays that look into (evolution of) gene regulatory networks could have helped to identify core transcription factors at work in the dorsal hindbrain of Ciona larva. The vertebrate cerebellum contains many cell types as well, and the cell type the authors think might be ancestral is actually not disclosed here. The comparative analysis with the mouse dataset was only discussed in relation to the (Purkinje) neurons.

Examples of aspects whose novelty is not clear and should be clarified

- The introduction mentions that the connectome is known. While Figures 1, 2 and 3 are very instructive on showing the connections, it is not immediately clear what is novel information beyond what was shown before with the full connectome. Are these figures derived from what has been shown in the connectome? Can it be made more clear in the text why and which additional experimental work was performed and what novel findings are derived?

- The way the results are written, notably providing reference to previous studies along with reference to specific figures (particularly obvious in the first two paragraphs), makes it hard to know what is novel and what is not. The phrasing should be modified to ensure that it conveys clear information about is replication of previous work and what is truly novel

Suggestions to clarify the evidence relative to the homology question or to get more depth in understanding the AMG neurons.

- Figure 4: panels A and B: please use the same pseudocolor for VGAT in both panels; the VGAT is supposed to look similar in both pictures, but it does not fully do that. It would help to delineate the brain areas in these images. Also, Lhx1/5 nor Otp are not exclusive to AMG. In what other cells they were expressed? Can these cells be annotated on the panels A and B (like it was done on D)?

- The authors created a merged sc dataset to study the transcriptional diversity and profile of the cells. The annotation of the clusters as well as the gene expression plots of the selected genes for in situ hybridisation/lineage marking is missing.

Where is evidence for the following statement in Figure 5, as there was no staining performed for these markers ?

"It is also unknown if either or both Otp and Lhx1/5 are necessary for inhibitory AMG development, although they all appear to be induced by the same FGF-dependent pathway (Fig. 5)."

## Author Response

Synthesis of Reviews:

Computational Neuroscience Model Code Accessibility Comments for Author (Required): N/A Synthesis Statement for Author (Required): Review Summary This manuscript has now been examined by two experts who both find the question of interest and agree on the overall quality of the methodological approaches and observations. However, they find the presented evidence insufficient to conclude that the AMG is homologous to, or a precursor of, the vertebrate cerebellum and as a result consider that the conclusions are overstated. They also note the novelty of some observations or approaches/tools is at times difficult to assess. Therefore, our main recommendations are to interpret the observations more neutrally, tone down the conclusions and make more explicit where findings or tools are novel in the text. More detailed assessments and specific recommendations and suggestions are provided here below.

Detailed assessments and specific comments This study investigates the central nervous system of the ascidian Ciona larva, focusing on the motor ganglion (MG) located posterior to the Neck region. The authors propose that the MG is divided into two functional domains along the dorsoventral axis and that the dorsal ascending motor ganglion interneurons (AMGs) may represent an ancestral form of the vertebrate cerebellum or a cerebellum-like structure. They used state-of-the-art tools to examine this hypothesis from multiple perspectives, comprehensively describe and functionally interrogate the connectome, gene expression, development and neurophysiological characteristics of the AMG neurons of the Ciona larva.

The observations and analyses of the AMGs are carefully performed, and the identification of distinct neuronal circuits and functions along the dorsoventral axis of the MG provides valuable insights indicating that the MG region posterior to the Neck in Ciona exhibits dorsoventral circuit differences, and that the dorsal AMG neurons function as a relay center for sensory input. The scRNA-seq analysis elucidating the AMG gene profile is also valuable. Nevertheless, the hypothesis that Ciona AMGs (a group of neurons) and the vertebrate cerebellum (a brain structure) share a common evolutionary origin-that is, true homology-remains inadequately supported. The developmental and genetic mechanisms differ substantially, making it difficult to view the AMG group as a cerebellar precursor. It is equally plausible that the AMG circuit represents a case of convergent evolution, in which a similar circuit motif was independently acquired. Because alternative explanations were less considered, the conclusions of this manuscript appear overstated. Moreover, what aspect of the findings or what tools/approaches are novel it is not always clear.

Therefore our main recommendations are to interpret the observations more neutrally and tone down the conclusions as well as make more explicit where findings or tools are novel in the text. Rather than emphasizing fragmentary similarities with the cerebellum, we believe that the study would be stronger if presented as a detailed description of the neural circuitry and gene expression patterns of the dorsal AMG neurons in Ciona rather than as evidence for cerebellar homology. Specific comments are listed here below and organized based on their nature and the importance.

As suggested, we have modified our manuscript to make our hypothesis more qualified. Nevertheless, our manuscript was submitted as a "Theory/New Concepts" article, which encourages authors to introduce new ideas. We feel that our article, which concerns speculative areas of evolution, fits these guidelines. While the manuscript does present our own original research, we also rely heavily on published data in formulating our hypothesis. We feel that our manuscript conforms well with the Theory/New Concepts category, which states that submissions are "not necessarily based on experimental data," and that they "question existing dogma in the field." Our hypothesis about deeper origins for the cerebellum is a main thrust, and it is this idea that questions existing dogma (previous publications posit that the cerebellum originated de novo in the vertebrates). Here is the difficult part of our data and hypothesis: the structure that we identified in Ciona (the AMG complex) does not have all of the attributes of a cerebellum, and our functional data confirms this (it is a sensory relay center - which is very different from the cerebellum). Yet, the AMG complex has enough cerebellum-like features to lead us to hypothesize that it and the cerebellum have a common origin. Our hypothesis is not that Ciona has a cerebellum, but rather that the structure from which the cerebellum arose in the vertebrates was already present at the tunicate/vertebrate split.

We offer this hypothesis based on multiple modes of data - gene expression, behavioral, anatomical, phylogenetic, and perhaps most unconventional to support a hypothesis such as ours, connectomic. With this approach, each piece of evidence presented and each argument put forward may only weakly support our hypothesis, but we believe that the SUM TOTAL of the evidence makes our hypothesis (our "New Concept") plausible, worthy of consideration, and even likely. Amongst these lines of evidence, we take a novel approach in the weight which we place on circuit homology, leveraging the power of the comparatively new field of connectomics. The readers may disagree with us as to whether this scenario is likely - this may hardly be surprising for a question that does, in fact, question current dogma in the field - but we believe positing this hypothesis is entirely justified given the entirety of the data we present.

While we understand our argument might be construed as stating that AMGs together constitute a cerebellum, or a cerebellar "prototype", we take pains not to make that claim. Our strongest claim is that the evidence point to a chordate ancestor already having a discrete processing center at the dorsal hindbrain; subsequent evolution of this region took very different trajectories after tunicates and vertebrates split, with the cerebellum arising independently in the vertebrates.

Specific comments Major issues relative to the conclusion - Neural Circuitry - The authors report that the "hindbrain" region posterior to the Neck contains distinct dorsal and ventral neural circuits and that the dorsal AMG functions as a relay center transmitting sensory information anteriorly. This finding is informative and contributes to our understanding of ascidian neurocircuitry. However, dorsoventral differences in neuronal distribution have already been reported in other regions of the Ciona CNS (Ryan et al., 2016, eLife), and such differences alone do not justify interpreting the dorsal region as a cerebellar prototype. Moreover, as the authors themselves acknowledge, the AMG circuitry differs in many respects from that of the cerebellum and more closely resembles that of a "cerebellum-like structure." The idea that the cerebellum evolved from a cerebellum-like structure remains hypothetical and is not yet supported by robust evidence.

1. We try not to say that the AMG complex is a cerebellum, rather we believe it shares homology to the structure which, much later, was elaborated into the cerebellum in the vertebrate lineage.

2. Yes, we agree that D-V differences do exist in the CNS of Ciona, we do not dispute this, such differences are also common in other chordates. For our hypothesis, a distinct dorsal hindbrain domain is necessary, but not sufficient, and represents only one factor among the data we present, whether anatomical, connectomic, gene expression-based, developmental origin, etc., that lead us to our hypothesis.

3. As noted, we point to the similarity of the AMG complex to cerebellum-like structures. The idea that the cerebellum might have evolved from a cerebellum-like structure is not our own, but has been previouslyhttps://www.zotero.org/google-docs/?GgOfKz. We consider this scenario and present it as hypothetical - this is the nature of evo-devo to present a most plausible hypothesis based on the evidence at hand.

- Interpretation of Developmental Position and Circuit Homology - The authors define the region anterior to the Neck (a constriction in the neural tube) as the midbrain and the posterior region as the hindbrain, and depict this scheme in their figures. This interpretation remains speculative and may mislead readers. As noted in the Introduction, homology of the midbrain and hindbrain between ascidians and vertebrates is only weakly supported by gene-expression patterns and relies primarily on functional correspondence inferred from connectomic data. The present study assumes the correctness of this pre-existing connectomic hypothesis and then further extends it to claim cerebellar homology . However, it is logically problematic to build a higher-level homology (with the cerebellum) upon an unverified lower-level homology (midbrain-hindbrain identity). While the Neck region indeed exhibits gene-expression similarities to the vertebrate midbrain-hindbrain boundary (MHB), MHB-like domains are also found in hemichordates lacking a central nervous system (Pani et al., 2012, Nature). Thus, MHB-like expression cannot by itself define adjacent midbrain and hindbrain regions. Furthermore, as the authors acknowledge, the key transcription factor Gbx, essential for hindbrain and cerebellar development in vertebrates, is absent from the ascidian genome. Response:

The correspondence between brain regions, fore, mid, hindbrain and spinal cord is based on multiple lines of evidence, not just gene expression patterns. In the manuscript, we discuss gene expression patterns that do not correspond directly between Ciona and vertebrates, e.g., the absence of Dmbx in the posterior sensory vesicle of Ciona. These are hardly surprising as each lineage has accumulated changes some 500 million plus years of independent evolution. Moreover, Ciona may represent a "streamlined" chordate body plan, with far fewer cells present during embryogenesis, a comparatively more determinative mode of development, and a rapidly evolving genome. That said, many of these patterns do correspond between Ciona and vertebrates. The field of evo-devo is replete with examples of tissues with unquestioned homology that nonetheless have divergent gene expression patterns (see below for further discussion).

Moreover, and with the evolutionary distances involved when comparing tunicates and vertebrates, we believe homology assignments cannot rely on diagnostic genes alone, but should encompass a broader range of evidence. So, for instance, we cite connectomic data showing sensory integration within the posterior sensory vesicle, pointing to functional similarity to the vertebrate tectum, which further lends support to this region's homology to the vertebrate midbrain. There is also extensive gene expression that supports this - for example the expression of Otx in the putative Ciona forebrain and midbrain homolog, but the expression of DMRThttps://www.zotero.org/google-docs/?fLW16fhttps://www.zotero.org/google-docs/?fLW16f. The putative midbrain is followed caudally by a structure called the "neck" region which, by position and gene expression has been equated by multiple authors to be homologous to the vertebrate midbrain/hindbrain organizer (MHB); organizing activity is likely dependent on Fgfhttps://www.zotero.org/google-docs/?OeqmtQhttps://www.zotero.org/google-docs/?OeqmtQ.

Posterior to the Ciona MHB is the so-called motor ganglion (MG), which shares many features with the vertebrate hindbrain. For example, in the manuscript, we point to Mauthner-like neurons (hindbrain neurons in vertebrates) - identified by their distinctive circuitry - and to neurons which show similarity to reticulospinal neurons (also hindbrain), including their gene expression and their descending projections to the caudal nerve cord where they innervate muscles. Moreover, we recently demonstrated that the elongated structure caudal to the MG has more homology to the spinal cord than previously appreciated. Significantly, we reported that the caudal region of the Ciona CNS does, in fact, contain functional motor neurons; the previously perceived lack of these neurons led some authors to claim that the MG in fact shared a composite homology of hindbrain/spinal cord, while the apparent lack of neurons in the tail left the caudal region a dead zone as far as homology assignment was concerned. For these more posterior regions of the Ciona CNS, similarity in axial Hox expression alone might somewhat complicate the homologies we've just outlined: Hox expression aligns between Ciona and vertebrates in some cases - Hox1 and Hox3 in the MG/hindbrain, Hox12 in the caudal tail/spinal cord - but in other cases does not - most notably Hox10 expression. Ikuta et al. have put forward the idea that the "disintegration" of the Hox cluster in Ciona (a single Hox cluster appears to have been split into two, and Hox members have been lost) has been accompanied by a diminished functionality of Hox genes during Ciona development https://www.zotero.org/google-docs/?cQkgR3. For example, markers for cholinergic and GABA/glycinergic neurons are unaffected by knockdowns of Hox1, 3 and 5.

In sum, we believe that the overall homology of brain regions - fore, mid, hind, MHB, HB, spinal cord - between Ciona and vertebrates is more than speculative and is backed by similarities beyond gene expression which, when we consider the sister relation between the groups, implied their homologies. We will point out again that determination of shared homology based on gene expression alone, although a common and, at times, a powerful approach, is also one that has its limitations. We feel that the Ciona connectome provides a wealth of data that can help resolve many of the issues.

There are other clear examples of homologous features between vertebrates and tunicates, e.g., heart, photoreceptors, notochord, that are unquestioned as to shared ancestry, but whose gene expression patterns are at times discordant. If we consider the notochord as an example, expression of a wnt5 ortholog in Ciona is reported as having faint expression in the notochord itself, in addition to expression in the surrounding mesodermal tissues of the tail as seen with a vertebrate ortholog of wnt5a in the zebrafish Danio rerio (pipetail/wnt5a). What's more, in another ascidian, Halocynthia roretzi, wnt5 is only observed in the notochord and not in surrounding mesoderm; Ciona and Halocynthia share a virtually identical pattern of cell division into tailbud stages of embryogenesis (and likely beyond); this difference in notochordal wnt5 gene expression, is at least an indication of the limitations of homology assignment based solely on diagnostic gene expression, a problem likely to increase over evolutionary distance.

- Ptf1a - The transcription factor Ptf1a, indispensable for Purkinje cell differentiation in the vertebrate cerebellum, is not expressed in the Ciona AMG. The authors' phylogenetic analysis (Fig. 4-1) argues that the known Ciona "Ptf1a" gene is actually an ortholog of the vertebrate Fer-3-like gene and that the true Ptf1a ortholog was lost in the tunicate lineage. However, the possibility that Ptf1a and Fer3-like genes diverged following the two rounds of whole-genome duplication (2R-WGD) in the vertebrate ancestor is not discussed. It is plausible that the ancestral Ptf1a/Fer3-like gene (including twist-like gene?) existed as a single copy in amphioxus and tunicates and that vertebrate Ptf1a and Fer3-like genes arose via duplication during 2R-WGD. If so, the Ciona "Ptf1a," even if clustered within the Fer3-like branch, could still represent the orthologous counterpart of vertebrate Ptf1a. Notably, the amphioxus Fer3-like gene was not included in the tree. The authors should re-analyze and clarify this issue.

Response:

We have included the amphioxus Fer3-like sequence ("lancelet fer3-like" on our new tree) and it clusters with the other Fer3-like genes, while amphioxus Ptf1a continues to group with the Ptf1a genes. This tree topology continues to support our initial idea that the gene called "Ptf1a" in Ciona is a Fer3-like gene, and a Ptf1a gene was lost in the tunicate lineage.

- Excitatory Neuron Differentiation and Atoh1 - Genes essential for the differentiation of excitatory granule cells in the vertebrate cerebellum, such as Atoh1, are not discussed. Although the authors show that AMG5 is excitatory, they acknowledge that it is cholinergic rather than glutamatergic, differing from cerebellar granule cells. They attribute this to a derived neurotransmitter change in tunicates, but if this is the case, additional molecular evidence-such as the expression of Atoh1-is required to support any proposed homology between AMG5 and granule cells. Without such evidence, it is difficult to regard AMG5 as sharing a common origin with cerebellar granule cells.

Response:

We have added language in the manuscript text to clarify that we do not claim that AMG5 and granule cells share a common origin.

- Gene-Expression Analysis - Using scRNA-seq and in situ hybridization, the authors identify the gene profile of the inhibitory AMG neurons, reporting expression of Lhx1/5, Otp, ASIC, VGAT, and HLH-2. This comprehensive analysis is valuable; however, none of these genes is specific to the cerebellum-they are broadly expressed in other CNS regions [see Points 1 and 2 in response below]. For example, Lhx1/5 is expressed in dorsal inhibitory interneurons of the spinal cord (Pillai et al., 2007, Development), and Otp is expressed in the hypothalamus (The Mouse Nervous System, 2012) and in inhibitory interneurons of the spinal cord (Worthy et al., 2024, eLife). Moreover, the vertebrate cerebellum arises exclusively from rhombomere 1, a segment that lacks expression of any Hox genes (Butts et al., 2014, Development). In contrast, Ciona AMGs express Hox10, and the authors even use the Hox10 cis-regulatory region to visualize AMG cells. While they argue that Hox10 expression represents a derived tunicate feature, their selective emphasis on genes that support cerebellar similarity (while attributing discordant patterns, such as Ptf1a absence or Hox10 expression, to lineage-specific modification) undermines the objectivity of the discussion [See Points 3 and 4 below]. A more systematic, unbiased method of cell type comparison between Ciona and other non-jawed and jawed vertebrate species would be necessary to solve the homology question - if at all it can be solved, given the small number of neurons.

Response:

1. It is true that none of these genes are specific to the cerebellum; we have stated in the text that they are expressed elsewhere in Ciona, as they are in vertebrates. It should be pointed out that nearly all genes used as cerebellum markers are also expressed elsewhere in other CNS regions. This includes cerebellin, AtOH, Ptf1a, and zebrin).

2. Moreover, while we point to commonality of expression patterns, patterns that are consistent with shared origins between the dorsal hindbrain regions of vertebrates and tunicates, we are not claiming that what is present in the dorsal hindbrain of Ciona (the AMGs) IS a cerebellum or even a cerebellum precursor. Rather, we believe the AMG complex of neurons a unique feature to the tunicates, but nonetheless, a feature which evidence points to a deeper chordate origin shared with the vertebrate cerebellum which, in turn, is a unique feature of the vertebrate lineage.

3. On the issue of Hox10: we do not attribute this as "discordant" without criteria. Hox10 is a posterior member of the Hox cluster, corresponding to the AbdB class Hox genes in insects (and also sharing more distant ancestry to Cdx of the ParaHox cluster). The colinearity of the Hox cluster - with more 5' members of the cluster expressed more posteriorly - is an ancient and conserved feature of bilaterians, revealing similar and ancient mechanisms to establish A-P polarity along the primary body axis. Ciona, on the other hand, has a genome relatively "under stress" and has lost strict clustering of its Hox genes https://www.zotero.org/google-docs/?HPIgfj. The lack of conformation in Ciona to the well conserved posterior expression of Hox10, as seen in posterior Hox expression in Drosophila and in Mus, can reasonably be inferred as unique to the Ciona lineage and an aberration from the broader pattern of colinearity seen for expression of Hox genes.

4. On Ptf1a-like, please see above item - in short, our phylogenetic analysis supports the idea that the Ciona Ptf1a-like is a Fer3-like gene and that the true Ptf1a-like ortholog has been lost.

- FGF-Signaling Inhibition Experiments - The authors treat embryos with the FGF-receptor inhibitor SU5402 and report loss of AMG cells, suggesting that AMG development depends on FGF signaling as in the vertebrate cerebellum. The SU5402 treatment effect in panels C-D of Figure 5 are a bit difficult to compare. How can we be sure the treatment did not affect the placement of the neurons? [see Point 1 in response below] Adding a more specific marker for AMG would more convincingly show absence of these neurons. [these are very distinct morphologically, we feel this is unnecessary and other markers would not be informative if the cells are "displaced"] Moreover, FGF signaling is essential in many brain regions, including the anterior neural ridge (ANR) of the telencephalon. Thus, FGF dependence alone is merely circumstantial evidence and cannot demonstrate developmental homology [but is consistent with it]. Furthermore, in Fig. 5D, not only the AMG domain but also adjacent VGAT-positive neurons exhibit marked morphological alterations. This raises the possibility that the observed effects reflect generalized developmental delay or nonspecific morphological defects in the CNS. Consequently, concluding that AMG development shares the same mechanism as cerebellar development seems overstated. In addition, SU5402 inhibits multiple receptor tyrosine kinases and is not specific to the FGF8 pathway. [For comments on last part of this paragraph, see Point 2] Response:

1. The ACINs are very distinct morphologically in their bifurcating axons; we feel an extra marker would be unnecessary and not informative if the cells are "displaced." 2. We have changed the text and are careful not to overstate the claim that is shares the exact mechanism as cerebellar development. In fact, the cerebellum, as a derived structure of the vertebrates appears to have a unique developmental progression, from rhombomeric lip and specification of Purkinje cells and of granular cells.

3. Nonetheless, we take the shared dependence on FGF of AMGs and cerebellum as yet another piece of evidence that deep shared origins for these dorsal hindbrain structures should be considered. Again, dependence on FGF alone does not unequivocally indicate one-to-one homology. Rather, we believe this evidence, along with other evidence presented, makes plausible the hypothesis that the cerebellum did not arise de novo from the vertebrate hindbrain, but instead can be traced to an antecedent in a pre-vertebrate ancestor, an ancestor also shared by Ciona.

- Spontaneous spiking is a feature of many neurons, also neurons outside of the cerebellum. It is not entirely clear how the measurements shown in Figure 6 contribute to the research question of cellular homology with cerebellar cells. The evidence that is presented here seems too preliminary to draw big conclusions from, and other types of analysis that can distinguish individual cells could bring deeper insight into firing characteristics, also upon stimulation.

There are many neurons which don't spike in this way, and we present data here showing that the GABAergic neurons of the AMG complex do show characteristic spiking.

We contrast these with Purkinje cells because - while we take pains to say AMGs are not Purkinje cells - Purkinje neurons may be a natural first point of reference because the two cell types share many similarities (inhibitory, anteriorly projecting, spiking). More generally, as we claim that AMGs/cerebellum may, in spite of their differences, share deep homology, this deep homology means that features of cerebellum are natural first things to look at when trying to characterize the AMGs when reasoning about the extent of this homology.

- The question whether dorsal hindbrain of Ciona (AMG cells) are evolutionary homologs of cerebellum in vertebrates has not been completely addressed, and while there are specific aspects that hint towards this relationship, these were not complete enough to be fully convincing. A more thorough investigation of the cell type homologies is missing, and other assays that look into (evolution of) gene regulatory networks could have helped to identify core transcription factors at work in the dorsal hindbrain of Ciona larva. The vertebrate cerebellum contains many cell types as well, and the cell type the authors think might be ancestral is actually not disclosed here. The comparative analysis with the mouse dataset was only discussed in relation to the (Purkinje) neurons.

To reiterate our hypothesis: we posit that the AMG complex and the cerebellum have origins in a structure that was found in their common ancestor. We are not hypothesizing that the AMGs are a cerebellum. We feel that this hypothesis is significant, because it can help to address questions regarding the advent of the cerebellum. While it is unquestionably a vertebrate invention, did it arise out of nothing? We hypothesize "no", there was already a dorsal hindbrain structure that gave rise to the cerebellum. We have performed a broader transcriptome analysis of our combined Ciona brain transcriptome data set to a mouse cerebellum transcriptome (this was in the Discussion). The results were inconclusive. We will include further mention of this in the manuscript.

Moreover, we don't claim that there are strict one-to-one homologies between AMGs and Purkinje cells or AMG5 and granule cells. Nonetheless, we use these vertebrate cell types as a point of comparison, as these are cells in the dorsal hindbrain where, in Ciona, the AMGs are also found. We hypothesize that while the AMGs and the cells of the cerebellum do not have one-to-one homologues, there may nevertheless be residual homology in gene expression carried forward from their common ancestors. Consistent with this, and with the enormous evolutionary distance between the tunicates and vertebrates, our preliminary transcriptome-wide analysis was not fruitful.

Examples of aspects whose novelty is not clear and should be clarified - The introduction mentions that the connectome is known. While Figures 1, 2 and 3 are very instructive on showing the connections, it is not immediately clear what is novel information beyond what was shown before with the full connectome. Are these figures derived from what has been shown in the connectome? Can it be made more clear in the text why and which additional experimental work was performed and what novel findings are derived? Response:

We agree that the connectome is published information and so not strictly novel. While reviews have been published describing major aspects ofhttps://www.zotero.org/google-docs/?nEJ9KA, no one has addressed or "mined" the connectome previously in this way, and in this context. The dorsal AMG neurons are predominantly ascending, the ventral MG are descending; these data are derived from the connectome, we have synthesized this data and pose evolutionary inferences based on circuit similarity.

Regarding whether these figures were "already there" - again we've mined data from the connectome which itself is a bare map/matrix, the data have not been presented this way previously. We feel that this is in keeping with the format of a "Theory/New Concepts" article.

- The way the results are written, notably providing reference to previous studies along with reference to specific figures (particularly obvious in the first two paragraphs), makes it hard to know what is novel and what is not. The phrasing should be modified to ensure that it conveys clear information about is replication of previous work and what is truly novel.

We have attempted to clarify this by including wording to indicate what has been previously published and what we found ourselves. For example, we use the first person plural "we" whenever it seems necessary to distinguish/clarify an experiment or observation as our own.

Suggestions to clarify the evidence relative to the homology question or to get more depth in understanding the AMG neurons.

- Figure 4: panels A and B: please use the same pseudocolor for VGAT in both panels; the VGAT is supposed to look similar in both pictures, but it does not fully do that. It would help to delineate the brain areas in these images. Also, Lhx1/5 nor Otp are not exclusive to AMG. In what other cells they were expressed? Can these cells be annotated on the panels A and B (like it was done on D)? These recommendations have been followed and the figures have been changed.

- The authors created a merged sc dataset to study the transcriptional diversity and profile of the cells. The annotation of the clusters as well as the gene expression plots of the selected genes for in situ hybridisation/lineage marking is missing.

Where is evidence for the following statement in Figure 5, as there was no staining performed for these markers ? "It is also unknown if either or both Otp and Lhx1/5 are necessary for inhibitory AMG development, although they all appear to be induced by the same FGF-dependent pathway (Fig. 5)." [check this]
